# Bayesian and E-Bayesian Estimations of Bathtub-Shaped Distribution under Generalized Type-I Hybrid Censoring

**DOI:** 10.3390/e23080934

**Published:** 2021-07-22

**Authors:** Yuxuan Zhang, Kaiwei Liu, Wenhao Gui

**Affiliations:** Department of Mathematics, Beijing Jiaotong University, Beijing 100044, China; 18271208@bjtu.edu.cn (Y.Z.); 18271071@bjtu.edu.cn (K.L.)

**Keywords:** bathtub-shaped distribution, Monte Carlo Markov Chain, E-Bayesian estimation, generalized Type-I hybrid censoring, Bayes estimation

## Abstract

For the purpose of improving the statistical efficiency of estimators in life-testing experiments, generalized Type-I hybrid censoring has lately been implemented by guaranteeing that experiments only terminate after a certain number of failures appear. With the wide applications of bathtub-shaped distribution in engineering areas and the recently introduced generalized Type-I hybrid censoring scheme, considering that there is no work coalescing this certain type of censoring model with a bathtub-shaped distribution, we consider the parameter inference under generalized Type-I hybrid censoring. First, estimations of the unknown scale parameter and the reliability function are obtained under the Bayesian method based on LINEX and squared error loss functions with a conjugate gamma prior. The comparison of estimations under the E-Bayesian method for different prior distributions and loss functions is analyzed. Additionally, Bayesian and E-Bayesian estimations with two unknown parameters are introduced. Furthermore, to verify the robustness of the estimations above, the Monte Carlo method is introduced for the simulation study. Finally, the application of the discussed inference in practice is illustrated by analyzing a real data set.

## 1. Introduction

### 1.1. Bathtub-Shaped Distribution

Chen [1] used the term ‘bathtub-shaped distribution’ to refer to a lifetime distribution that possesses an increasing or bathtub-shaped hazard function with two parameters. As it could depict the lifetimes for multiple mechanical and electrical products, this distribution is widely used in practice. There have been several further investigations into the study of bathtub-shaped distribution. Before [1] named the two-parameter lifetime distribution with the above characteristics of a hazard rate function as a bathtub-shaped distribution, a reliability distribution with a bathtub-shaped failure rate was proposed by [2], and ref. [3] employed an effective method to analyze data with a bathtub failure rate by introducing the exponentiated Weibull family.

Furthermore, ref. [4] considered the Bayes estimations and estimates of two unknown parameters based on the maximum likelihood method under a bathtub-shaped distribution. Additionally, a considerable amount of literature has been published on estimations under bathtub-shaped distribution based on a censoring scheme. The authors in [5] focused on the maximum likelihood method to calculate point estimators and derived an exact joint confidence region and confidence interval of parameters based on a progressively Type-II censoring sample. The researchers in [6] investigated the Fisher information matrix, maximum likelihood estimates, and confidence intervals for unknown parameters under hybrid censored data.

The probability density function (pdf) and cumulative distribution function of a bathtub-shaped distribution take the forms, respectively,    

(1) $$\begin{array}{c} f\left(x;\lambda ,\beta \right)=\lambda \beta x^{\beta -1}\exp\left\{\lambda \left(-e^{x^{\beta }}+1\right)+x^{\beta }\right\},\lambda ,\beta >0,\;x>0, \end{array}$$

(2) $$\begin{array}{c} F\left(x;\lambda ,\beta \right)=-\exp\{\lambda \left(-e^{x^{\beta }}+1\right)\}+1,\lambda ,\beta >0,\;x>0. \end{array}$$

The reliability function and hazard rate function are given by

(3) $$\begin{array}{c} R\left(t\right)=\exp\{\lambda \left(-e^{t^{\beta }}+1\right)\},\lambda ,\beta >0,\;t>0, \end{array}$$

(4) $$\begin{array}{c} h\left(x\right)=\lambda \beta e^{x^{\beta }}x^{\beta -1},\lambda ,\beta >0,\;x>0. \end{array}$$

**Figure 1:**
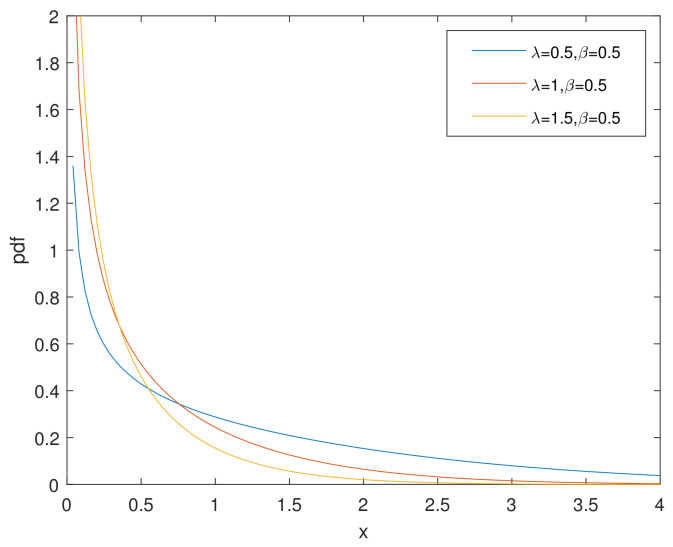
pdf of CHD when β=0.5.

**Figure 2:**
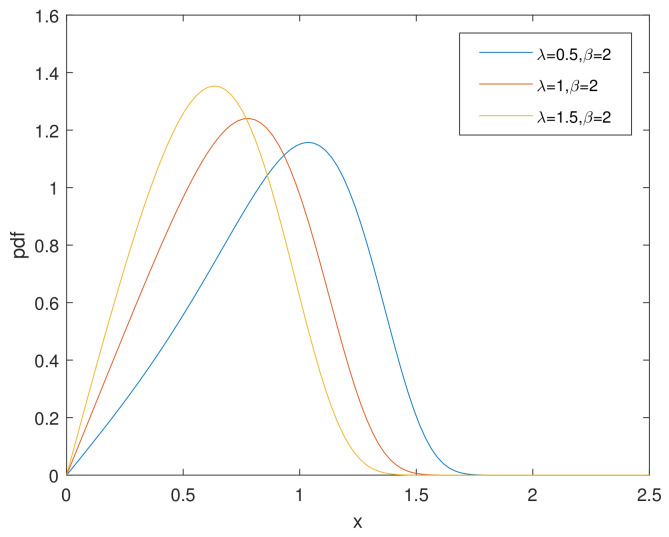
pdf of CHD when β=2.

**Figure 3:**
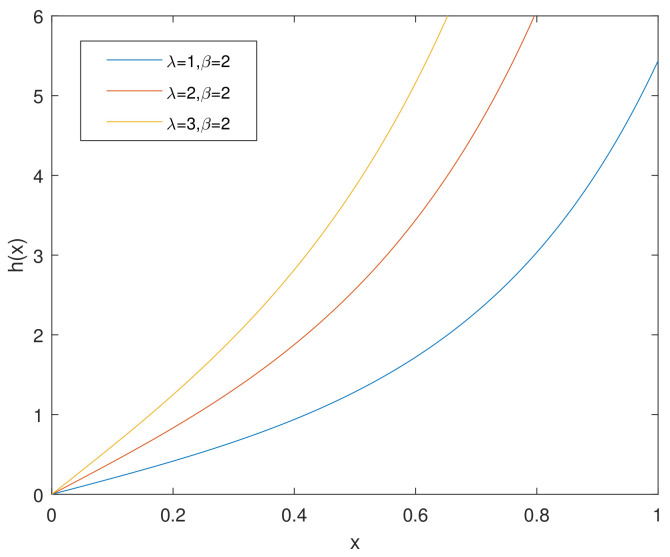
h(x) of CHD when λ≤1,β=2.

**Figure 4:**
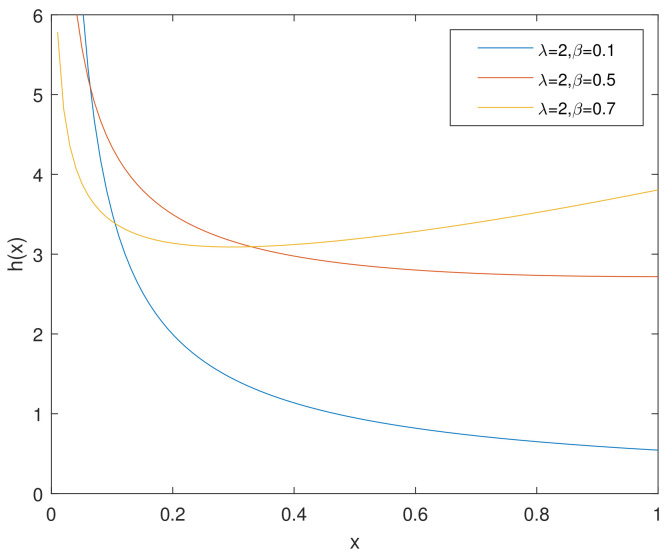
h(x) of CHD when β<1,λ=2.

**Figure 5:**
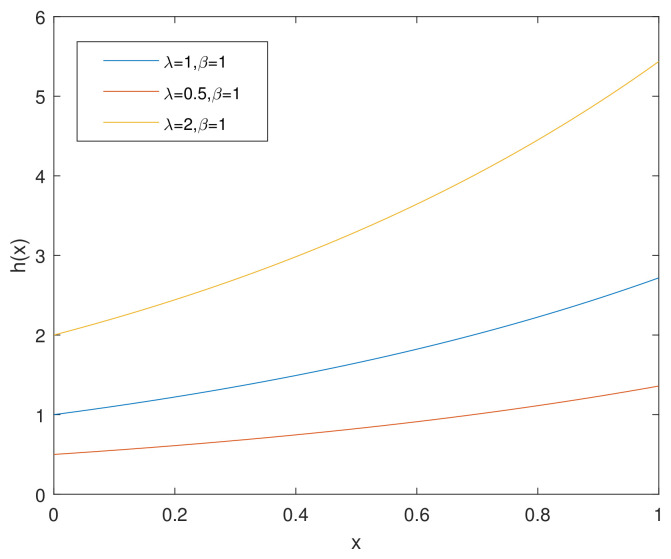
h(x) of CHD when β=1.

For simplicity, we denote the bathtub-shaped distribution as CHD with the parameters (λ,β). The parameter λ has few influence on the shape of its hazard rate function; however, β makes a difference to the hazard rate function instead. h(x) is bathtub-shaped if the shape parameter β<1, otherwise it appears as an increasing function. In particular, according to [7], when β=1, CHD reduces to the exponential power distribution. Figure 1 and Figure 2 and Figure 3, Figure 4 and Figure 5 show the pdf and h(x) of a bathtub-shaped distribution, respectively.

### 1.2. Generalized Hybrid Type-I Censoring Scheme

There is no doubt that estimations based on complete samples are more accurate. However, it is inevitable to use censoring for lifetime experiments due to time constraints and expense reduction. Type-I and Type-II censoring are usually considered as two fundamental methods to conduct lifetime experiments, where we terminate these experiments at a certain time point or upon the occurrence of a certain number of failures. With the rapid development of science and technology, products have higher reliability and longer life spans, resulting in a longer time of life-testing to obtain sufficient failure samples.

In order to cut down the life-testing duration, ref. [8] carried out a hybrid Type-I censoring scheme that could be considered as a combination of those two fundamental censoring schemes discussed above. Under this scheme, lifetime experiments operate after a specific point of time and the number of failures is pre-fixed. As long as either of these occurs, the test will be terminated. However, this scheme also has limitations as it has a possibility that extremely few failures occur before the pre-determined time. As a result, it may be impractical to make statistical inferences under such a scheme.

In order to overcome this disadvantage and improve the efficiency of estimators in the life-testing experiment as well as to guarantee that a certain number of failures appear before the end of the experiment as well as saving the time of testing and the cost resulted from failures of units, ref. [9] introduced a generalized hybrid Type-I censoring scheme. Generalized hybrid Type-I censoring assures a minimum number of failures, which could mitigate the short back that exists in hybrid Type-I censoring. For simplicity, we denote this as Type-I GHCS.

We assume that $X_{1:n}$, $\cdots ,X_{n:n}$ are *n* ordered observations of failure lifetime. *r* and *T* are fixed in advance, where *r* represents the ideal number of failures and *T* is the timepoint. These three mentioned censoring models can be expressed as

- Type-I censoring: terminate at *T*.
- Hybrid Type-I censoring: terminate at $T^{*}=\min\left\{X_{r:n},T\right\}$.
- Type-I GHCS: terminate at $T_{*}=\max\left\{X_{k:n},\min\left\{X_{r:n},T\right\}\right\}$ where k<r<n and *k* is the minimum acceptable number of failures fixed before the experiment.

In this article, we focus on Type-I GHCS, and it can be divided into three cases:

- Case I: $\left\{X_{1:n}<\cdots <X_{k:n}\right\}$, when $X_{k:n}>T$.
- Case II: $\left\{X_{1:n}<\cdots <X_{r:n}\right\}$, when $X_{r:n}<T$.
- Case III: $\left\{X_{1:n}<\cdots <X_{d:n}\right\}$, when $X_{k:n}<T<X_{r:n}$.

Ref. [9] introduced exact likelihood estimation of exponential lifetime distribution based on GHCS. Ref. [10] discussed inferential issues under hybrid censoring schemes and presented details on developments regarding generalized hybrid censoring. Ref. [11] studied estimations of a single parameter from a Burr-X distribution under Type-I GHCS. Furthermore, ref. [12] applied an acceptance sampling plan under Weibull distribution under GHCS.

Suppose that $x_{i:n}$ is the *i*-th failure time based on samples from a bathtub-shaped distrubtion under Type-I GHCS. The likelihood function is shown as

(5) $$\begin{array}{c} L\left(\lambda \right)=\left\{\begin{array}{cc} \frac{n!}{(n-k)!}\lambda^{k}\beta^{k}\exp\left\{\left(n-k\right)\lambda W_{k}\right\}\prod_{i=1}^{k}\left[{x_{i:n}}^{\beta -1}\left(1-W_{i}\right)e^{\lambda W_{i}}\right], & D<k\\ \frac{n!}{(n-D)!}\lambda^{D}\beta^{D}\exp\left\{\left(n-D\right)\lambda W_{T}\right\}\prod_{i=1}^{D}\left[{x_{i:n}}^{\beta -1}\left(1-W_{i}\right)e^{\lambda W_{i}}\right], & k\leq D<r\\ \frac{n!}{(n-r)!}\lambda^{r}\beta^{r}\exp\left\{\left(n-r\right)\lambda W_{r}\right\}\prod_{i=1}^{r}\left[{x_{i:n}}^{\beta -1}\left(1-W_{i}\right)e^{\lambda W_{i}}\right], & D\geq r \end{array}\right., \end{array}$$

where $W_{i}=-{e^{x_{i:n}}}^{\beta }+1$, $W_{k}=-{e^{x_{k:n}}}^{\beta }+1$, $W_{T}=-{e^{T}}^{\beta }+1$, $W_{r}=-{e^{x_{r:n}}}^{\beta }+1$, and *D* represents the number of failures before timepoint *T*.

According to (5), the likelihood function is translated into the following form,

(6) $$\begin{array}{c} L\left(\lambda \right)\propto \lambda^{n}\exp\left(-\lambda Q\right), \end{array}$$

where

(7) $$\begin{array}{c} Q=\left\{\begin{array}{cc} -\sum_{i=1}^{k}W_{i}, & D<k\\ -\sum_{i=1}^{D}W_{i}, & k\leq D<r\\ -\sum_{i=1}^{r}W_{i}, & D\geq r \end{array}\right.. \end{array}$$

The MLE of parameter λ can be derived by the equation below, $\frac{\partial L}{\partial \lambda }=n\lambda^{n-1}\exp\left(-\lambda Q\right)-Q\lambda^{n}\exp\left(-\lambda Q\right)=0$.

From the equation above, the MLE of λ is obtained as

(8) $$\begin{array}{c} \hat{\lambda }=\frac{n}{Q}. \end{array}$$

By the same method, the MLE of parameter β can be derived by the equation,

(9) $$\begin{array}{c} \frac{\partial L}{\partial \beta }=\left\{\begin{array}{cc} \frac{k}{\beta }+\sum_{i=1}^{k}\ln\left(x_{i:n}\right)\left(1+{x_{i:n}}^{\beta }\right)+\frac{k}{W_{k}}\ln\left(x_{k:n}\right)e^{{x_{k:n}}^{\beta }}{x_{k:n}}^{\beta }, & D<k\\ \frac{D}{\beta }+\sum_{i=1}^{T}\ln\left(x_{i:n}\right)\left(1+{x_{i:n}}^{\beta }\right)+\frac{D}{W_{T}}\ln\left(x_{T:n}\right)e^{{x_{T:n}}^{\beta }}{x_{T:n}}^{\beta }, & k\leq D<r\\ \frac{r}{\beta }+\sum_{i=1}^{r}\ln\left(x_{i:n}\right)\left(1+{x_{i:n}}^{\beta }\right)+\frac{r}{W_{r}}\ln\left(x_{r:n}\right)e^{{x_{r:n}}^{\beta }}{x_{r:n}}^{\beta }, & D\geq r \end{array}\right.=0. \end{array}$$

Previous studies based on bathtub-shaped distribution have always dealt with censored samples under a typical statistical inference method—maximum likelihood estimation for instance. Ref. [13] investigated the estimation problems of unknown parameters, reliability, hazard rate functions, and their approximate confidence intervals under the maximum likelihood method and credible intervals under the Bayesian estimation method.

However, there has been no previous study to coalesce a generalized Type-I hybrid censoring scheme with a bathtub-shaped distribution under the E-Bayesian method. Therefore, our main purpose is to investigate estimations of the scale parameters and reliability function of bathtub-shaped distribution under E-Bayesian and Bayesian methods based on a generalized Type-I hybrid censoring scheme with the presupposition that the shape parameter is known.

The remainder of this paper is organized as follows. Section 2 investigates Bayesian estimations against squared error and LINEX loss functions under Type-I GHCS. Section 3 compares the E-Bayesian estimations derived from three different prior distributions. Section 4 introduces Bayesian and E-Bayesian estimations with two unknown parameters. Section 5 establishes the results of a Monte Carlo simulation study with the Metropolis–Hasting algorithm for the purpose of evaluating the effects of different methods and prior distributions on estimators. Section 5 presents a numerical example from a real data set for the purpose of examining the theoretical inference discussed above.

## 2. Bayesian Estimation

Bayesian estimation measures the uncertainties of unknown parameters by connecting the prior information from a random sample with certain distributions. Prior distributions as well as loss functions affect the accuracy of estimation under the Bayesian method. In this section, under two different loss functions, we assume the parameter β is known and calculate the estimation of scale parameter λ and the reliability function under a bathtub-shaped distribution based on Type-I GHCS. Then, we derive Bayesian estimations.

First, we suppose that λ follows the gamma conjugate prior distribution given by:

(10) $$\begin{array}{c} \pi \left(\lambda \right)=\frac{b^{a}}{\Gamma (a)}\lambda^{a-1}e^{-b\lambda },\;a,b>0. \end{array}$$

On the basis of the Bayesian method, we multiply (10) by (5) to obtain the posterior distribution of λ

(11) $$\begin{array}{c} \pi \left(\lambda |\tilde{x}\right)=\left\{\begin{array}{cc} \kappa^{-1}\lambda^{k+a-1}e^{-b\lambda }\prod_{i=1}^{k}e^{\lambda W_{i}}\exp\left[\lambda \left(n-k\right)W_{k})\right], & D<k\\ \kappa^{-1}\lambda^{D+a-1}e^{-b\lambda }\prod_{i=1}^{D}e^{\lambda W_{i}}\exp\left[\lambda \left(n-D\right)W_{T})\right], & k\leq D<r\\ \kappa^{-1}\lambda^{r+a-1}e^{-b\lambda }\prod_{i=1}^{r}e^{\lambda W_{i}}\exp\left[\lambda \left(n-r\right)W_{r})\right], & D\geq r \end{array}\right., \end{array}$$

where $\tilde{x}=\left(x_{1:n},\cdots ,x_{n:n}\right)$ and κ could be written in the following form

(12) $$\begin{array}{c} \kappa =\left\{\begin{array}{cc} \begin{array}{c} \int_{0}^{+\infty }\;\lambda^{k+a-1}e^{-b\lambda }\prod_{i=1}^{k}e^{\lambda W_{i}}\exp\left[\lambda \left(n-k\right)W_{k})\right]\;d\lambda \end{array}, & D<k\\ \begin{array}{c} \int_{0}^{+\infty }\;\lambda^{D+a-1}e^{-b\lambda }\prod_{i=1}^{D}e^{\lambda W_{i}}\exp\left[\lambda \left(n-D\right)W_{T})\right]\;d\lambda \end{array}, & k\leq D<r\\ \begin{array}{c} \int_{0}^{+\infty }\;\lambda^{r+a-1}e^{-b\lambda }\prod_{i=1}^{r}e^{\lambda W_{i}}\exp\left[\lambda \left(n-r\right)W_{r})\right]\;d\lambda \end{array}, & D\geq r \end{array}\right., \end{array}$$

First, we adopt a symmetrical loss function called the squared error (SE) loss function, which lays weight equally on overestimation and underestimation. Based on this loss function, Bayesian estimations are equivalent to the posterior means, which could be obtained to be, respectively,

(13) $$\begin{array}{c} {\hat{\lambda }}_{BS}=E\left(\lambda |\tilde{x}\right)=\begin{array}{c} \int_{0}^{+\infty }\;\lambda \pi (\lambda |\tilde{x})\;d\lambda \end{array}=\left\{\begin{array}{cc} \frac{K+a}{-\sum_{i=1}^{k}W_{i}-\left(n-k\right)W_{k}+b}, & D<k\\ \frac{D+a}{-\sum_{i=1}^{D}W_{i}-\left(n-D\right)W_{T}+b}, & k\leq D<r\\ \frac{r+a}{-\sum_{i=1}^{r}W_{i}-\left(n-r\right)W_{r}+b}, & D\geq r \end{array}\right.. \end{array}$$

(14) $$\begin{array}{c} \begin{array}{cc} {\hat{R(t)}}_{BS} & =E(R\left(t\right)|\tilde{x})\\ & =\begin{array}{c} \int_{0}^{+\infty }\;R\left(t\right)\pi (\lambda |\tilde{x})\;d\lambda \end{array}\\ & =\left\{\begin{array}{cc} {\left(\frac{b-\sum_{i=1}^{k}W_{i}-\left(n-k\right)W_{k}}{-\sum_{i=1}^{k}W_{i}-\left(n-k\right)W_{k}+b+P^{*}}\right)}^{K+a}, & D<k\\ {\left(\frac{b-\sum_{i=1}^{D}W_{i}-\left(n-D\right)W_{T}}{-\sum_{i=1}^{D}W_{i}-\left(n-D\right)W_{T}+b+P^{*}}\right)}^{D+a}, & k\leq D<r\\ {\left(\frac{b-\sum_{i=1}^{r}W_{i}-\left(n-r\right)W_{r}}{-\sum_{i=1}^{r}W_{i}-\left(n-r\right)W_{r}+b+P^{*}}\right)}^{r+a}, & D\geq r \end{array}\right. \end{array}, \end{array}$$

where $P^{*}=e^{t^{\beta }}-1$.

Secondly, we consider a LINEX loss function with an asymmetric shape, which is commonly used in practice as it is more realistic to illustrate the loss according to ratio. The Bayesian estimation of λ against the LINEX loss function can be given by

(15) $$\begin{array}{c} \begin{array}{cc} {\hat{\lambda }}_{BL} & =-\frac{1}{h}\ln E(e^{-h\lambda }|\tilde{x})=-\frac{1}{h}\ln\begin{array}{c} \int_{0}^{+\infty }\;e^{-h\lambda }\pi (\lambda |\tilde{x})\;d\lambda \end{array}\\ & =\left\{\begin{array}{cc} \frac{K+a}{h}\ln\left(1+\frac{h}{b-\sum_{i=1}^{k}W_{i}-\left(n-k\right)W_{k}}\right), & D<k\\ \frac{a+D}{h}\ln\left(1+\frac{h}{b-\sum_{i=1}^{D}W_{i}-\left(n-D\right)W_{T}}\right), & k\leq D<r\\ \frac{a+r}{h}\ln\left(1+\frac{h}{b-\sum_{i=1}^{r}W_{i}-\left(n-r\right)W_{r}}\right), & D\geq r \end{array}\right. \end{array}. \end{array}$$

Similarly, the Bayesian estimator of R(t), under a LINEX loss function, is derived in the following form:

(16) $$\begin{array}{c} \begin{array}{cc} {\hat{R(t)}}_{BL} & =-\frac{1}{h}lnE(e^{-hR(t)}|\tilde{x})\\ & =\left\{\begin{array}{cc} -\frac{1}{h}\ln\left[\sum_{i=1}^{+\infty }\frac{{\left(-h\right)}^{i}}{\Gamma (i)}{\left(\frac{K}{K+iP^{*}}\right)}^{b}\right], & D<k\\ -\frac{1}{h}\ln\left[\sum_{i=1}^{+\infty }\frac{{\left(-h\right)}^{i}}{\Gamma (i)}{\left(\frac{D}{D+iP^{*}}\right)}^{b}\right], & k\leq D<r\\ -\frac{1}{h}\ln\left[\sum_{i=1}^{+\infty }\frac{{\left(-h\right)}^{i}}{\Gamma (i)}{\left(\frac{r}{r+iP^{*}}\right)}^{b}\right], & D\geq r \end{array}\right. \end{array}. \end{array}$$

## 3. E-Bayesian Estimation

Considering that the prior information may be deficient, the E-Bayesian method could be used to settle the uncertainty by introducing a class of priors. The authors in [14] demonstrated that, based on a specified prior distribution, the purpose of the E-Bayesian method is to estimate unknown parameters or to predict values of a sequence of random variables.

Under SE and LINEX loss functions, we derive E-Bayesian estimators of λ and the reliability function. Additionally, for the purpose of perceiving the effects of prior distributions on E-Bayesian estimations, three different prior distributions are considered. The authors in [15] indicated that, in order to ensure that π(λ|a,b) is decreasing, the hyper parameters *a* and *b* are chosen. In the case of λ, the derivative of π(λ) could be obtained as

$$\begin{array}{c} \frac{d\pi (\lambda )}{d\lambda }=\frac{b^{a}}{\Gamma (a)}\lambda^{a-2}e^{-b\lambda }\left[(a-1)-b\lambda \right]. \end{array}$$

It is apparent that the prior distribution π(λ) is a decreasing function in λ when 0<a<1 and b>0. Assume that the bivariate density function in which *a* and *b* are independent is

$$\begin{array}{c} \pi \left(a,b\right)=\pi_{1}\left(a\right)\pi_{2}\left(b\right). \end{array}$$

According to [16], when the parameter *a* is given, with the increase of *b*, the tailed prior distribution will be thinner, which would likely reduce the robustness of Bayesian estimations. Therefore, *b* is selected to be smaller than a pre-determined constant *c*. In this case, for parameter λ and R(t), the E-Bayesian estimations are obtained as

(17) $$\begin{array}{c} {\hat{\lambda }}_{EB}=E\left({\hat{\lambda }}_{BS}|\tilde{x}\right)=\int_{0}^{1}\int_{0}^{c}{\hat{\lambda }}_{BS}\left(a,b\right)\pi (a,b)dadb, \end{array}$$

(18) $$\begin{array}{c} {\hat{R(t)}}_{EB}=E\left({\hat{R(t)}}_{BS}\right)\left|\tilde{x}\right)=\int_{0}^{1}\int_{0}^{c}{\hat{R(t)}}_{BS}\left(a,b\right)\pi (a,b)dadb. \end{array}$$

Next, for the purpose of exploring the influence of a prior distribution on an estimator under the E-Bayesian method, we derive the estimates under three different prior distributions. These three different prior distributions are selected as follows:

(19) $$\begin{array}{c} \begin{array}{cc} \pi_{1}\left(a,b\right) & =\frac{1}{cB(u,v)}a^{u-1}{\left(1-a\right)}^{v-1},\\ \pi_{2}\left(a,b\right) & =\frac{2(c-b)}{c^{2}B\left(u,v\right)}a^{u-1}{\left(1-a\right)}^{v-1}\\ \pi_{3}\left(a,b\right) & =\frac{2b}{c^{2}B\left(u,v\right)}a^{u-1}{\left(1-a\right)}^{v-1}, \end{array},0<b<c,0<a<1, \end{array}$$

where B(u,v) is the beta function. $\pi_{1}\left(a,b\right)$ is a constant in *b*, while $\pi_{2}\left(a,b\right)$ is a decreasing function in *b* and $\pi_{3}\left(a,b\right)$ is an increasing function in *b*.

### 3.1. E-Bayesian Estimations Based on SE Loss Function

Based on the SE loss function, the E-Bayesian estimations of λ with the prior distribution $\pi_{1}\left(a,b\right)$ can be obtained from (13), (17) and (19) as

(20) $$\begin{array}{c} \begin{array}{cc} {\hat{\lambda }}_{EBS1} & =\int_{0}^{1}\int_{0}^{c}{\hat{\lambda }}_{BS}\left(a,b\right)\pi_{1}\left(a,b\right)dadb\\ & =\frac{1}{cB(u,v)}\begin{array}{c} \int_{0}^{1}\int_{0}^{c}\;\left(\frac{K+a}{P+b}\right)a^{u-1}{\left(1-a\right)}^{v-1}\;dbda \end{array}\\ & =\left\{\begin{array}{cc} \frac{1}{c}\left(K+\frac{u}{u+v}\right)\ln\left(1+\frac{P_{k}}{c}\right), & D<k\\ \frac{1}{c}\left(D+\frac{u}{u+v}\right)\ln\left(1+\frac{P_{T}}{c}\right), & k\leq D<r\\ \frac{1}{c}\left(r+\frac{u}{u+v}\right)\ln\left(1+\frac{P_{r}}{c}\right), & D\geq r \end{array}\right., \end{array} \end{array}$$

where $P_{k}=\sum_{i=1}^{k}W_{i}-\left(n-k\right)W_{k}$, $P_{T}=\sum_{i=1}^{D}W_{i}-\left(n-D\right)W_{T}$, $P_{r}=\sum_{i=1}^{r}W_{i}-\left(n-r\right)W_{r}$.

Likewise, the E-Bayesian estimations of λ under $\pi_{2}\left(a,b\right)$ and $\pi_{3}\left(a,b\right)$ could be written, respectively, in the following forms:

(21) $$\begin{array}{c} \begin{array}{c} {\hat{\lambda }}_{EBS2}=\left\{\begin{array}{cc} \frac{2}{c}\left(K+\frac{u}{u+v}\right)\left[\left(1+\frac{P_{k}}{c}\right)ln(1+\frac{c}{P_{k}})-1\right], & D<k\\ \frac{2}{c}\left(D+\frac{u}{u+v}\right)\left[\left(1+\frac{P_{T}}{c}\right)ln(1+\frac{c}{P_{T}})-1\right], & k\leq D<r\\ \frac{2}{c}\left(r+\frac{u}{u+v}\right)\left[\left(1+\frac{P_{r}}{c}\right)ln(1+\frac{c}{P_{r}})-1\right], & D\geq r \end{array}\right. \end{array}. \end{array}$$

(22) $$\begin{array}{c} {\hat{\lambda }}_{EBS3}=\left\{\begin{array}{cc} \frac{2}{c}\left(K+\frac{u}{u+v}\right)\left[1-\frac{P_{k}}{c}\ln\left(1+\frac{c}{P_{k}}\right)\right], & D<k\\ \frac{2}{c}\left(D+\frac{u}{u+v}\right)\left[1-\frac{P_{T}}{c}\ln\left(1+\frac{c}{P_{T}}\right)\right], & k\leq D<r\\ \frac{2}{c}\left(r+\frac{u}{u+v}\right)\left[1-\frac{P_{r}}{c}\ln\left(1+\frac{c}{P_{r}}\right)\right], & D\geq r \end{array}\right.. \end{array}$$

### 3.2. E-Bayesian Estimations Based on a LINEX Loss Function

Under a LINEX loss function, the E-Bayesian estimation of λ with the prior distribution $\pi_{1}\left(a,b\right)$ can be obtained from (14), (17), and (19) as

(23) $$\begin{array}{c} \begin{array}{cc} & {\hat{\lambda }}_{EBL1}\\ = & \int_{0}^{1}\int_{0}^{c}{\hat{\lambda }}_{BL}\pi_{1}\left(a,b\right)dadb\\ = & \frac{1}{chB(u,v)}\begin{array}{c} \int_{0}^{1}\int_{0}^{c}\;\left(m+a\right)a^{u-1}{\left(1-a\right)}^{v-1}\ln(1+\frac{h}{b+P})\;dbda \end{array}\\ = & \left\{\begin{array}{cc} \frac{1}{ch}\left(K+\frac{u}{u+v}\right)\left[c\ln\left(1+\frac{h}{c+P_{k}}\right)+\left(P_{k}+h\right)\ln\left(1+\frac{h}{P_{k}+h}\right)-P_{k}\ln\left(1+\frac{c}{P_{k}}\right)\right], & D<k\\ \frac{1}{ch}\left(D+\frac{u}{u+v}\right)\left[c\ln\left(1+\frac{h}{c+P_{T}}\right)+\left(P_{T}+h\right)\ln\left(1+\frac{h}{P_{T}+h}\right)-P_{T}\ln\left(1+\frac{c}{P_{T}}\right)\right], & k\leq D<r\\ \frac{1}{ch}\left(r+\frac{u}{u+v}\right)\left[c\ln\left(1+\frac{h}{c+P_{r}}\right)+\left(P_{r}+h\right)\ln\left(1+\frac{h}{P_{r}+h}\right)-P_{r}\ln\left(1+\frac{c}{P_{r}}\right)\right], & D\geq r \end{array}\right. \end{array}. \end{array}$$

Under the same method, the E-Bayesian estimations of λ under $\pi_{2}\left(a,b\right)$ and $\pi_{3}\left(a,b\right)$ could be written, respectively, as

(24) $$\begin{array}{c} \begin{array}{cc} & {\hat{\lambda }}_{EBL2}\\ = & \int_{0}^{1}\int_{0}^{c}{\hat{\lambda }}_{BL}\pi_{2}\left(a,b\right)dadb\\ = & \left\{\begin{array}{cc} \left(K+\frac{u}{u+v}\right)\left[\frac{\ln(\frac{P_{k}+h}{P_{k}})}{h}-\frac{{\left(P_{k}+c\right)}^{2}}{c^{2}h}\ln\left(\frac{P_{k}+c}{P_{k}}\right)+\frac{{\left(P_{k}+c+h\right)}^{2}}{c^{2}h}\ln\left(\frac{P_{k}+h+c}{P_{k}+h}\right)-\frac{1}{c}\right], & D<k\\ \left(D+\frac{u}{u+v}\right)\left[\frac{\ln(\frac{P_{t}+h}{P_{t}})}{h}-\frac{{\left(P_{T}+c\right)}^{2}}{c^{2}h}\ln\left(\frac{P_{t}+c}{P_{T}}\right)+\frac{{\left(P_{T}+c+h\right)}^{2}}{c^{2}h}\ln\left(\frac{P_{T}+h+c}{P_{T}+h}\right)-\frac{1}{c}\right], & k\leq D<r\\ \left(r+\frac{u}{u+v}\right)\left[\frac{\ln(\frac{P_{r}+h}{P_{r}})}{h}-\frac{{\left(P_{r}+c\right)}^{2}}{c^{2}h}\ln\left(\frac{P_{r}+c}{P_{r}}\right)+\frac{{\left(P_{r}+c+h\right)}^{2}}{c^{2}h}\ln\left(\frac{P_{r}+h+c}{P_{r}+h}\right)-\frac{1}{c}\right], & D\geq r \end{array}\right. \end{array}. \end{array}$$

(25) $$\begin{array}{c} \begin{array}{cc} & {\hat{\lambda }}_{EBL3}\\ = & \int_{0}^{1}\int_{0}^{c}{\hat{\lambda }}_{BL}\pi_{3}\left(a,b\right)dadb\\ = & \left\{\begin{array}{cc} \left(K+\frac{u}{u+v}\right)\left[\frac{1}{h}\ln\left(1+\frac{h}{c+P_{k}}\right)+\frac{{P_{k}}^{2}}{c^{2}h}\ln\left(1+\frac{c}{P_{k}}\right)-\frac{{\left(P_{k}+h\right)}^{2}}{c^{2}h}\ln\left(1+\frac{c}{P_{k}+h}\right)+\frac{1}{c}\right], & D<k\\ \left(D+\frac{u}{u+v}\right)\left[\frac{1}{h}\ln\left(1+\frac{h}{c+P_{T}}\right)+\frac{{P_{T}}^{2}}{c^{2}h}\ln\left(1+\frac{c}{P_{T}}\right)-\frac{{\left(P_{T}+h\right)}^{2}}{c^{2}h}\ln\left(1+\frac{c}{P_{T}+h}\right)+\frac{1}{c}\right], & k\leq D<r\\ \left(r+\frac{u}{u+v}\right)\left[\frac{1}{h}\ln\left(1+\frac{h}{c+P_{r}}\right)+\frac{{P_{r}}^{2}}{c^{2}h}\ln\left(1+\frac{c}{P_{r}}\right)-\frac{{\left(P_{r}+h\right)}^{2}}{c^{2}h}\ln\left(1+\frac{c}{P_{r}+h}\right)+\frac{1}{c}\right], & D\geq r \end{array}\right. \end{array}. \end{array}$$

### 3.3. E-Bayesian Estimations of R(t)

The E-Bayesian estimation of R(t) under an SE loss function can be derived from (15), (17) and (19) by using the prior distribution $\pi_{1}\left(a,b\right)$,

(26) $$\begin{array}{c} \begin{array}{cc} {\hat{R(t)}}_{EBS1}= & \int_{0}^{1}\int_{0}^{c}{\hat{R}}_{BS}\left(t\right)\pi_{1}\left(a,b\right)dadb\\ = & \left\{\begin{array}{cc} \frac{1}{c}\int_{0}^{c}\;{\left(1+\frac{P^{*}}{b+P^{*}}\right)}^{-k}F_{1:1}\left(u,u+v;\ln\left(\frac{b+P_{k}}{b+P_{k}+P*}\right)\right)\;db, & D<k\\ \frac{1}{c}\int_{0}^{c}\;{\left(1+\frac{P^{*}}{b+P^{*}}\right)}^{-D}F_{1:1}\left(u,u+v;\ln\left(\frac{b+P_{T}}{b+P_{T}+P*}\right)\right)\;db, & k\leq D<r\\ \frac{1}{c}\int_{0}^{c}\;{\left(1+\frac{P^{*}}{b+P^{*}}\right)}^{-r}F_{1:1}\left(u,u+v;\ln\left(\frac{b+P_{r}}{b+P_{r}+P*}\right)\right)\;db, & D\geq r \end{array}\right. \end{array}, \end{array}$$

where $F_{1:1}\left(.,.;.\right)$ is the generalized hypergeometic function. For more details, one can refer to [17].

Under $\pi_{2}\left(a,b\right)$ and $\pi_{3}\left(a,b\right)$, the E-Bayesian estimations of R(t) are written in the following forms under the same method.

(27) $$\begin{array}{c} \begin{array}{cc} {\hat{R(t)}}_{EBS2} & =\int_{0}^{1}\int_{0}^{c}{\hat{R}}_{BS}\left(t\right)\pi_{2}\left(a,b\right)dadb\\ & =\left\{\begin{array}{cc} \frac{2}{c^{2}}\int_{0}^{c}\;\left(c-b\right){\left(1+\frac{P^{*}}{b+P_{k}}\right)}^{-k}F_{1:1}\left(u,u+v;\ln\left(\frac{b+P_{k}}{b+P_{k}+P*}\right)\right)\;db, & D<k\\ \frac{2}{c^{2}}\int_{0}^{c}\;\left(c-b\right){\left(1+\frac{P^{*}}{b+P_{T}}\right)}^{-D}F_{1:1}\left(u,u+v;\ln\left(\frac{b+P_{T}}{b+P_{T}+P*}\right)\right)\;db, & k\leq D<r\\ \frac{2}{c^{2}}\int_{0}^{c}\;\left(c-b\right){\left(1+\frac{P^{*}}{b+P_{r}}\right)}^{-r}F_{1:1}\left(u,u+v;\ln\left(\frac{b+P_{r}}{b+P_{r}+P*}\right)\right)\;db, & D\geq r \end{array}\right. \end{array}. \end{array}$$

(28) $$\begin{array}{c} \begin{array}{cc} {\hat{R(t)}}_{EBS3} & =\int_{0}^{1}\int_{0}^{c}{\hat{R}}_{BS}\left(t\right)\pi_{3}\left(a,b\right)dadb\\ & =\left\{\begin{array}{cc} \frac{2}{c^{2}}\int_{0}^{c}\;b{\left(1+\frac{P^{*}}{b+P_{k}}\right)}^{-k}F_{1:1}\left(u,u+v;\ln\left(\frac{b+P_{k}}{b+P_{k}+P*}\right)\right)\;db, & D<k\\ \frac{2}{c^{2}}\int_{0}^{c}\;b{\left(1+\frac{P^{*}}{b+P_{T}}\right)}^{-D}F_{1:1}\left(u,u+v;\ln\left(\frac{b+P_{T}}{b+P_{T}+P*}\right)\right)\;db, & k\leq D<r\\ \frac{2}{c^{2}}\int_{0}^{c}\;b{\left(1+\frac{P^{*}}{b+P_{r}}\right)}^{-r}F_{1:1}\left(u,u+v;\ln\left(\frac{b+P_{r}}{b+P_{r}+P*}\right)\right)\;db, & D\geq r \end{array}\right. \end{array}. \end{array}$$

Under a LINEX loss function, the E-Bayesian estimation of R(t) with different prior distributions can be obtained from (16), (17) and (19) as follows,

(29) $$\begin{array}{c} {\hat{R(t)}}_{EBLi}=\int_{0}^{1}\int_{0}^{c}{\hat{R}}_{BL}\left(t\right)\pi_{i}\left(a,b\right)dadb,i=1,2,3. \end{array}$$

The integrals in (26), (27), and (28) are not in simple closed forms. Additionally, the integrals in (29) are also infeasible to derive. Thus, to further evaluate E-Bayesian estimates of R(t), the numerical technique should be introduced.

## 4. Estimation with Two Unknown Parameters

### 4.1. Bayesian Estimation

In this section, we assume that λ and β are independent and follow a gamma prior distribution:

(30) $$\begin{array}{c} \pi \left(\lambda \right)=\frac{{b_{1}}^{a_{1}}}{\Gamma (a_{1})}\lambda^{a_{1}-1}e^{-b_{1}\lambda },\;a_{1},b_{1}>0, \end{array}$$

(31) $$\begin{array}{c} \pi \left(\beta \right)=\frac{{b_{2}}^{a_{2}}}{\Gamma (a_{2})}\beta^{a_{2}-1}e^{-b_{2}\beta },\;a_{2},b_{2}>0. \end{array}$$

Thus, the joint prior distribution is obtained as

(32) $$\begin{array}{c} \pi \left(\lambda ,\beta \right)=\frac{{b_{1}}^{a_{1}}}{\Gamma (a_{1})}\frac{{b_{2}}^{a_{2}}}{\Gamma (a_{2})}\lambda^{a_{1}-1}\beta^{a_{2}-1}e^{-(b_{1}\lambda +b_{2}\beta )},\;a_{1},b_{1},a_{2},b_{2}>0. \end{array}$$

On the basis of Bayesian method, we multiply (32) by (5) to obtain the joint posterior distribution

(33) $$\begin{array}{c} \pi \left(\lambda |\tilde{x}\right)=\left\{\begin{array}{cc} \kappa^{-1}\lambda^{k+a_{1}-1}\beta^{k+a_{2}-1}e^{-(b_{1}\lambda +b_{2}\beta )}\prod_{i=1}^{k}e^{\lambda W_{i}}\exp\left[\lambda \left(n-k\right)W_{k})\right], & D<k\\ \kappa^{-1}\lambda^{D+a_{1}-1}\beta^{D+a_{2}-1}e^{-(b_{1}\lambda +b_{2}\beta )}\prod_{i=1}^{D}e^{\lambda W_{i}}\exp\left[\lambda \left(n-D\right)W_{T})\right], & k\leq D<r\\ \kappa^{-1}\lambda^{r+a_{1}-1}\beta^{r+a_{2}-1}e^{-(b_{1}\lambda +b_{2}\beta )}\prod_{i=1}^{r}e^{\lambda W_{i}}\exp\left[\lambda \left(n-r\right)W_{r})\right], & D\geq r \end{array}\right., \end{array}$$

where $\tilde{x}=\left(x_{1:n},\cdots ,x_{n:n}\right)$ and κ could be written in the following form

(34) $$\begin{array}{c} \kappa =\left\{\begin{array}{cc} \begin{array}{c} \int_{0}^{+\infty }\int_{0}^{+\infty }\;\lambda^{k+a_{1}-1}\beta^{k+a_{2}-1}e^{-(b_{1}\lambda +b_{2}\beta )}\prod_{i=1}^{k}e^{\lambda W_{i}}\exp\left[\lambda \left(n-k\right)W_{k})\right]\;d\lambda d\beta \end{array}, & D<k\\ \begin{array}{c} \int_{0}^{+\infty }\int_{0}^{+\infty }\;\lambda^{D+a_{1}-1}\beta^{D+a_{2}-1}e^{-(b_{1}\lambda +b_{2}\beta )}\prod_{i=1}^{D}e^{\lambda W_{i}}\exp\left[\lambda \left(n-D\right)W_{T})\right]\;d\lambda d\beta \end{array}, & k\leq D<r\\ \begin{array}{c} \int_{0}^{+\infty }\int_{0}^{+\infty }\;\lambda^{r+a_{1}-1}\beta^{r+a_{2}-1}e^{-(b_{1}\lambda +b_{2}\beta )}\prod_{i=1}^{r}e^{\lambda W_{i}}\exp\left[\lambda \left(n-r\right)W_{r})\right]\;d\lambda d\beta \end{array}, & D\geq r \end{array}\right.. \end{array}$$

Similarly, we could obtain the Bayesian estimations of two unknown parameters and R(t) under SE and LINEX loss functions.

(35) $$\begin{array}{c} \begin{array}{cc} {\hat{\lambda }}_{{BS}_{2}} & =E\left(\lambda |\tilde{x}\right)=\begin{array}{c} \int_{0}^{+\infty }\int_{0}^{+\infty }\;\lambda \pi (\lambda ,\beta |\tilde{x})\;d\lambda d\beta \end{array}\\ & =\left\{\begin{array}{cc} \frac{1}{\kappa }\int_{0}^{+\infty }\int_{0}^{+\infty }\;\lambda^{k+a_{1}}\beta^{k+a_{2}-1}e^{-(b_{1}\lambda +b_{2}\beta )}\prod_{i=1}^{k}e^{\lambda W_{i}}\exp\left[\lambda \left(n-k\right)W_{k})\right]\;d\lambda d\beta , & D<k\\ \frac{1}{\kappa }\int_{0}^{+\infty }\int_{0}^{+\infty }\;\lambda^{D+a_{1}}\beta^{D+a_{2}-1}e^{-(b_{1}\lambda +b_{2}\beta )}\prod_{i=1}^{D}e^{\lambda W_{i}}\exp\left[\lambda \left(n-D\right)W_{T})\right]\;d\lambda d\beta , & k\leq D<r\\ \frac{1}{\kappa }\int_{0}^{+\infty }\int_{0}^{+\infty }\;\lambda^{r+a_{1}}\beta^{r+a_{2}-1}e^{-(b_{1}\lambda +b_{2}\beta )}\prod_{i=1}^{r}e^{\lambda W_{i}}\exp\left[\lambda \left(n-r\right)W_{r})\right]\;d\lambda d\beta , & D\geq r \end{array}\right. \end{array}. \end{array}$$

(36) $$\begin{array}{c} \begin{array}{cc} {\hat{\lambda }}_{{BL}_{2}} & =-\frac{1}{h}\ln E(e^{-h\lambda }|\tilde{x})=-\frac{1}{h}\ln\begin{array}{c} \int_{0}^{+\infty }\int_{0}^{+\infty }\;e^{-h\lambda }\pi (\lambda ,\beta |\tilde{x})\;d\lambda d\beta \end{array}\\ & =\left\{\begin{array}{cc} \frac{1}{\kappa }\int_{0}^{+\infty }\int_{0}^{+\infty }\;\lambda^{k+a_{1}-1}\beta^{k+a_{2}-1}e^{-(\left(b_{1}+h\right)\lambda +b_{2}\beta )}\prod_{i=1}^{k}e^{\lambda W_{i}}\exp\left[\lambda \left(n-k\right)W_{k})\right]\;d\lambda d\beta , & D<k\\ \frac{1}{\kappa }\int_{0}^{+\infty }\int_{0}^{+\infty }\;\lambda^{D+a_{1}-1}\beta^{D+a_{2}-1}e^{-(\left(b_{1}+h\right)\lambda +b_{2}\beta )}\prod_{i=1}^{D}e^{\lambda W_{i}}\exp\left[\lambda \left(n-D\right)W_{T})\right]\;d\lambda d\beta , & k\leq D<r\\ \frac{1}{\kappa }\int_{0}^{+\infty }\int_{0}^{+\infty }\;\lambda^{r+a_{1}-1}\beta^{r+a_{2}-1}e^{-(\left(b_{1}+h\right)\lambda +b_{2}\beta )}\prod_{i=1}^{r}e^{\lambda W_{i}}\exp\left[\lambda \left(n-r\right)W_{r})\right]\;d\lambda d\beta , & D\geq r \end{array}\right. \end{array}. \end{array}$$

(37) $$\begin{array}{c} \begin{array}{cc} {\hat{\beta }}_{{BS}_{2}} & =E\left(\beta |\tilde{x}\right)=\begin{array}{c} \int_{0}^{+\infty }\int_{0}^{+\infty }\;\beta \pi (\lambda ,\beta |\tilde{x})\;d\lambda d\beta \end{array}\\ & =\left\{\begin{array}{cc} \frac{1}{\kappa }\int_{0}^{+\infty }\int_{0}^{+\infty }\;\lambda^{k+a_{1}-1}\beta^{k+a_{2}}e^{-(b_{1}\lambda +b_{2}\beta )}\prod_{i=1}^{k}e^{\lambda W_{i}}\exp\left[\lambda \left(n-k\right)W_{k})\right]\;d\lambda d\beta , & D<k\\ \frac{1}{\kappa }\int_{0}^{+\infty }\int_{0}^{+\infty }\;\lambda^{D+a_{1}-1}\beta^{D+a_{2}}e^{-(b_{1}\lambda +b_{2}\beta )}\prod_{i=1}^{D}e^{\lambda W_{i}}\exp\left[\lambda \left(n-D\right)W_{T})\right]\;d\lambda d\beta , & k\leq D<r\\ \frac{1}{\kappa }\int_{0}^{+\infty }\int_{0}^{+\infty }\;\lambda^{r+a_{1}-1}\beta^{r+a_{2}}e^{-(b_{1}\lambda +b_{2}\beta )}\prod_{i=1}^{r}e^{\lambda W_{i}}\exp\left[\lambda \left(n-r\right)W_{r})\right]\;d\lambda d\beta , & D\geq r \end{array}\right. \end{array}. \end{array}$$

(38) $$\begin{array}{c} \begin{array}{cc} {\hat{\beta }}_{{BL}_{2}} & =-\frac{1}{h}\ln E(e^{-h\beta }|\tilde{x})=-\frac{1}{h}\ln\begin{array}{c} \int_{0}^{+\infty }\int_{0}^{+\infty }\;e^{-h\beta }\pi (\lambda ,\beta |\tilde{x})\;d\lambda d\beta \end{array}\\ & =\left\{\begin{array}{cc} \frac{1}{\kappa }\int_{0}^{+\infty }\int_{0}^{+\infty }\;\lambda^{k+a_{1}-1}\beta^{k+a_{2}-1}e^{-(b_{1}\lambda +\left(b_{2}+h\right)\beta )}\prod_{i=1}^{k}e^{\lambda W_{i}}\exp\left[\lambda \left(n-k\right)W_{k})\right]\;d\lambda d\beta , & D<k\\ \frac{1}{\kappa }\int_{0}^{+\infty }\int_{0}^{+\infty }\;\lambda^{D+a_{1}-1}\beta^{D+a_{2}-1}e^{-(b_{1}\lambda +\left(b_{2}+h\right)\beta )}\prod_{i=1}^{D}e^{\lambda W_{i}}\exp\left[\lambda \left(n-D\right)W_{T})\right]\;d\lambda d\beta , & k\leq D<r\\ \frac{1}{\kappa }\int_{0}^{+\infty }\int_{0}^{+\infty }\;\lambda^{r+a_{1}-1}\beta^{r+a_{2}-1}e^{-(b_{1}\lambda +\left(b_{2}+h\right)\beta )}\prod_{i=1}^{r}e^{\lambda W_{i}}\exp\left[\lambda \left(n-r\right)W_{r})\right]\;d\lambda d\beta , & D\geq r \end{array}\right. \end{array}. \end{array}$$

(39) $$\begin{array}{c} \begin{array}{cc} {\hat{R(t)}}_{{BS}_{2}} & =E(R\left(t\right)|\tilde{x})\\ & =\begin{array}{c} \int_{0}^{+\infty }\int_{0}^{+\infty }\;R\left(t\right)\pi (\lambda ,\beta |\tilde{x})\;d\lambda d\beta \end{array}\\ & =\left\{\begin{array}{cc} \frac{1}{\kappa }\int_{0}^{+\infty }\int_{0}^{+\infty }\;\frac{\lambda^{k+a_{1}-1}\beta^{k+a_{2}-1}}{e^{b_{1}\lambda +b_{2}\beta +he^{\lambda (1-e^{t^{\beta }})}}}\prod_{i=1}^{k}e^{\lambda W_{i}}\exp\left[\lambda \left(n-k\right)W_{k})\right]\;d\lambda d\beta , & D<k\\ \frac{1}{\kappa }\int_{0}^{+\infty }\int_{0}^{+\infty }\;\frac{\lambda^{D+a_{1}-1}\beta^{D+a_{2}-1}}{e^{b_{1}\lambda +b_{2}\beta +he^{\lambda (1-e^{t^{\beta }})}}}\prod_{i=1}^{D}e^{\lambda W_{i}}\exp\left[\lambda \left(n-D\right)W_{T})\right]\;d\lambda d\beta , & k\leq D<r\\ \frac{1}{\kappa }\int_{0}^{+\infty }\int_{0}^{+\infty }\;\frac{\lambda^{r+a_{1}-1}\beta^{r+a_{2}-1}}{e^{b_{1}\lambda +b_{2}\beta +he^{\lambda (1-e^{t^{\beta }})}}}\prod_{i=1}^{r}e^{\lambda W_{i}}\exp\left[\lambda \left(n-r\right)W_{r})\right]\;d\lambda d\beta , & D\geq r \end{array}\right. \end{array}. \end{array}$$

We could not directly calculate these above integrals in simple closed form, but the approximate Bayesian estimators could be derived under Lindley’s aprroximation. For more details, one can refer to [18].

### 4.2. E-Bayesian Estimation

According to E-Bayesian estimation with unknown parameter λ, we select the prior distributions for parameter λ and β as follows,

(40) $$\begin{array}{c} \begin{array}{cc} \pi_{11}\left(\lambda |a_{1},b_{1}\right) & =\frac{1}{c_{1}B\left(u_{1},v_{1}\right)}{a_{1}}^{u_{1}-1}{\left(1-a_{1}\right)}^{v_{1}-1},\\ \pi_{12}\left(\lambda |a_{1},b_{1}\right) & =\frac{2(c_{1}-b_{1})}{{c_{1}}^{2}B\left(u_{1},v_{1}\right)}{a_{1}}^{u_{1}-1}{\left(1-a_{1}\right)}^{v_{1}-1}\\ \pi_{13}\left(\lambda |a_{1},b_{1}\right) & =\frac{2b_{1}}{{c_{1}}^{2}B\left(u_{1},v_{1}\right)}{a_{1}}^{u_{1}-1}{\left(1-a_{1}\right)}^{v_{1}-1}, \end{array},0<b_{1}<c_{1},0<a_{1}<1, \end{array}$$

(41) $$\begin{array}{c} \begin{array}{cc} \pi_{21}\left(\beta |a_{2},b_{2}\right) & =\frac{1}{c_{2}B\left(u_{2},v_{2}\right)}{a_{2}}^{u_{2}-1}{\left(1-a_{2}\right)}^{v_{2}-1},\\ \pi_{22}\left(\beta |a_{2},b_{2}\right) & =\frac{2(c_{2}-b_{2})}{{c_{2}}^{2}B\left(u_{2},v_{2}\right)}{a_{2}}^{u_{2}-1}{\left(1-a_{2}\right)}^{v_{2}-1}\\ \pi_{23}\left(\beta |a_{2},b_{2}\right) & =\frac{2b_{2}}{{c_{2}}^{2}B\left(u_{2},v_{2}\right)}{a_{2}}^{u_{2}-1}{\left(1-a_{2}\right)}^{v_{2}-1}, \end{array},0<b_{2}<c_{2},0<a_{2}<1, \end{array}$$

where $B(u_{1},v_{1})$ and $B(u_{2},v_{2})$ are beta functions. Under an SE loss function, the E-Bayesian estimations can be obtained from (40), (41), (35), and (37) as,

(42) $$\begin{array}{c} {\hat{\lambda }}_{EBSi}=\int_{0}^{1}\int_{0}^{c_{1}}{\hat{\lambda }}_{BS}\pi_{i}\left(a_{1},b_{1}\right)da_{1}db_{1}, \end{array}$$

(43) $$\begin{array}{c} {\hat{\beta }}_{EBSi}=\int_{0}^{1}\int_{0}^{c_{1}}{\hat{\beta }}_{BS}\pi_{i}\left(a_{1},b_{1}\right)da_{1}db_{1} \end{array}$$

Under a LINEX loss function, the E-Bayesian estimations can be obtained from (40), (41), (36), and (38) as,

(44) $$\begin{array}{c} {\hat{\lambda }}_{ELSi}=\int_{0}^{1}\int_{0}^{c_{2}}{\hat{\lambda }}_{BL}\pi_{i}\left(a_{2},b_{2}\right)da_{2}db_{2}, \end{array}$$

(45) $$\begin{array}{c} {\hat{\beta }}_{EBSi}=\int_{0}^{1}\int_{0}^{c_{2}}{\hat{\beta }}_{BL}\pi_{i}\left(a_{2},b_{2}\right)da_{2}db_{2}. \end{array}$$

Similarly, we could use the MCMC method to compute E-Bayesian estimations.

## 5. MCMC Method and Simulation Study

According to the Markov Chain Monte Carlo algorithm, we could approximate the integral when it cannot be generated explicitly for multidimensional problems. Therefore, the MCMC algorithm is a widely used and effective method to obtain samples from complex posterior distributions. We apply the Monte Carlo simulation under Type-I GHCS in this section to compute E-Bayesian estimates of λ and R(t) against different prior distributions and loss functions.

According to (11), the full conditional posterior probability density function of the parameter λ is written as,

(46) $$\begin{array}{c} \pi^{*}\left(\lambda |x\right)=\left\{\begin{array}{cc} \lambda^{k+a-1}e^{-b\lambda }\prod_{i=1}^{k}e^{\lambda W_{i}}\exp\left[\lambda \left(n-k\right)W_{k})\right], & D<k\\ \lambda^{D+a-1}e^{-b\lambda }\prod_{i=1}^{D}e^{\lambda W_{i}}\exp\left[\lambda \left(n-D\right)W_{T})\right], & k\leq D<r\\ \lambda^{r+a-1}e^{-b\lambda }\prod_{i=1}^{r}e^{\lambda W_{i}}\exp\left[\lambda \left(n-r\right)W_{r})\right], & D\geq r \end{array}\right.. \end{array}$$

As the conditional posterior PDF of λ is complex, we introduce the MCMC method to obtain random samples by considering a normal distribution as the proposal distribution.

The MCMC approach is shown in Algorithm 1. We could refer to [19,20] for more details regarding the implementation of MCMC algorithm.
**Algorithm 1** MCMC algorithm.1:Set the initial value $\lambda^{\left(0\right)}$ be equal to the MLE $\hat{\lambda }$.2:Set v=1.3:**repeat**4: Take $N(\hat{\lambda },Var\left(\hat{\lambda }\right))$ as the proposal distribution and generate $\lambda^{\left(*\right)}$ from it at iteration *v*.5: Obtain the samples under Type-I GHCS from Uniform(0,1) distribution.6: Compute the acceptance probability: $p\left(\lambda^{\left(v-1\right)}|\lambda^{\left(*\right)}\right)=\min\left[\frac{\pi^{*}\left(\lambda^{\left(*\right)}|\tilde{x}\right)}{\pi^{*}\left(\lambda^{\left(v-1\right)}|\tilde{x}\right)},1\right]$.7: **if**  u < p  **then**8:  $\lambda^{\left(v\right)}=\lambda^{\left(*\right)}$;9: **else**10:  $\lambda^{\left(v\right)}=\lambda^{\left(v-1\right)}$;11:** end if**12: Compute the value of $R^{\left(v\right)}\left(t\right)$ as $R^{\left(v\right)}\left(t\right)=\exp\left\{\lambda^{\left(v\right)}\left(1-e^{x^{\beta }}\right)\right\}$.13:**until** v=N14:Set $\delta =\frac{N}{10}$ as the burn-in period.15:Based on the SE loss function,           ${\hat{\lambda }}_{BS}=\frac{1}{N-\delta }\sum_{v=\delta +1}^{N}\lambda^{\left(v\right)}$,         ${\hat{R(t)}}_{BS}=\frac{1}{N-\delta }\sum_{v=\delta +1}^{N}R^{\left(v\right)}\left(t\right)$.16:Based on the LINEX loss function,          ${\hat{\lambda }}_{BL}=-\frac{1}{h}\ln\left[\frac{1}{N-\delta }\sum_{v=\delta +1}^{N}e^{-h\lambda^{\left(v\right)}}\right]$,         ${\hat{R(t)}}_{BL}=-\frac{1}{h}\ln\left[\frac{1}{N-\delta }\sum_{v=\delta +1}^{N}e^{-hR^{\left(v\right)}\left(t\right)}\right]$.17:Compute the credible intervals of Bayesian and E-Bayesian estimations
$$\begin{array}{c} \left(\lambda_{\left[N\left(\frac{\gamma }{2}\right)\right]},\lambda_{\left[N\left(1-\frac{\gamma }{2}\right)\right]}\right) \end{array}$$
and
$$\begin{array}{c} (R{\left(t\right)}_{\left[N\left(\frac{\gamma }{2}\right)\right]},R{\left(t\right)}_{\left[N\left(1-\frac{\gamma }{2}\right)\right]}). \end{array}$$
where [a]={[a]∈Z,[a]≤a}, γ is the significant level, and *N* is the amount of draws.

For Bayesian and E-Bayesian methods, for the purpose of evaluating and comparing the performance of estimators against different loss functions, we perform simulation comparisons with data derived from different scenarios. We assume that parameter β is fixed as constant 1. Given a particular value to *c*, *a* and *b* can be obtained according to (19). The algorithm of generating and analyzing data based on Type-I GHCS under the bathtub-shaped distribution is shown in Algorithm 2.
**Algorithm 2** The algorithm of generating and analyzing data.1:Generate samples under Type-I GHCS from CHD(λ,β):
i) Generate *n* independent variables $U=(U_{1},U_{2},\cdots ,U_{n})$ from Uniform(0,1) distribution.
1.For given r,k,T, set *m* as,
(47)$$m=\left\{\begin{array}{cc} k & D<k\\ r & k\leq D<r\\ d & D\geq r \end{array}\right..$$2.Generalized Type-I hybrid censored samples $X=(X_{1:n}$, ⋯,$X_{m:n})$ are derived by the inverse function method: $X={\left[\ln(1-\frac{\ln(1-U)}{\lambda })\right]}^{\frac{1}{\beta }}.$2:Repeat the simulation for N= 11,000 times.3:Discard δ= 1000 iterations (the burn-in) in each chains.4:Use the values of parameter λ from the sampler to generate Bayesian and E-Bayesian estimates.5:Compare the effectiveness of different methods according to the mean square error MSE of λ and R(t), where
$$\begin{array}{c} MSE\left(\hat{\lambda }\right)=\frac{1}{N-\delta }\sum_{v=\delta +1}^{N}{\left({\hat{\lambda }}_{v}-\lambda \right)}^{2}, \end{array}$$
$$\begin{array}{c} MSE\left(\hat{R}\left(t\right)\right)=\frac{1}{N-\delta }\sum_{v=\delta +1}^{N}{\left({\hat{R}\left(t\right)}_{v}-R\left(t\right)\right)}^{2}. \end{array}$$Steps 2–4 are corresponding to steps 1–16 in Algorithm 1.

Under different (n,(r,k),T,β), we draw samples from every simulation, and values of MLE are computed. Additionally, we obtain the MLE and Bayesian estimation under each simulation.

In order to facilitate the simulation, according to [21], we take the special case

(48) $$\begin{array}{c} \pi \left(a,b\right)=\frac{1}{c}, \end{array}$$

where c>0.

In this case, the E−MSE of $\hat{\lambda }$ could be obtained as,

(49) $$\begin{array}{c} E-MSE\left({\hat{\lambda }}_{EBS}\right)=\int_{0}^{1}\int_{0}^{c}MSE({\hat{\lambda }}_{BS})\pi (a,b)dadb=\left\{\begin{array}{cc} \frac{2k+1}{2Q(Q+c)}\; & D<k\\ \frac{2D+1}{2Q(Q+c)}\; & k\leq D<r\\ \frac{2r+1}{2Q(Q+c)}\; & D\geq r \end{array}\right.. \end{array}$$

(50) $$\begin{array}{c} \begin{array}{cc} E-MSE({\hat{\lambda }}_{EBL}) & =\int_{0}^{1}\int_{0}^{c}MSE({\hat{\lambda }}_{BL})\pi (a,b)dadb\\ & =\left\{\begin{array}{c} \frac{{\left(a+k\right)}^{2}}{h^{2}}\int_{0}^{1}\int_{0}^{c}\frac{h^{2}\left(a+k+1\right)}{a+k}\ln\frac{1}{{\left(Q+b\right)}^{2}}+\frac{2h}{b+Q}\ln\frac{Q+b}{h+Q+b}+{\left(\ln\frac{Q+b}{h+Q+b}\right)}^{2}dadb\;\\ \frac{{\left(a+D\right)}^{2}}{h^{2}}\int_{0}^{1}\int_{0}^{c}\frac{h^{2}\left(a+D+1\right)}{a+D}\ln\frac{1}{{\left(Q+b\right)}^{2}}+\frac{2h}{b+Q}\ln\frac{Q+b}{h+Q+b}+{\left(\ln\frac{Q+b}{h+Q+b}\right)}^{2}dadb\;\\ \frac{{\left(a+r\right)}^{2}}{h^{2}}\int_{0}^{1}\int_{0}^{c}\frac{h^{2}\left(a+r+1\right)}{a+r}\ln\frac{1}{{\left(Q+b\right)}^{2}}+\frac{2h}{b+Q}\ln\frac{Q+b}{h+Q+b}+{\left(\ln\frac{Q+b}{h+Q+b}\right)}^{2}dadb\; \end{array}\right.. \end{array} \end{array}$$

The sample size (n,(r,k),T) is fixed from the data under Type-I GHCS and is set to n=50,80,120 with three sets of fixed numbers (r,k)=(40,30),(60,40),(90,60) presented respectively for each size. Simultaneously, for the purpose of studying the reliability function under a bathtub-shaped distribution, we set *T* as T=0.2,0.4.

Based on the tabulated estimates and the mean square errors of the estimations whose statistical inference processes are computed from software R, the following conclusion can be drawn from Table 1, Table 2, Table 3, Table 4, Table 5 and Table 6 and Table 7, Table 8 and Table 9, in which the true value of λ takes 4.37 and 2.5, respectively, and E−MSE represents the mean square error of the E-Bayesian estimations. Table 1, Table 2, Table 3 and Table 4 show the results of estimations when λ=4.37,β=1 in detail. Table 5, Table 6, Table 7, Table 8 and Table 9 guarantee the robustness of the conclusion. See more details on the MCMC outputs in Appendix A.

1. Both estimates are close to their theoretical values under different methods and loss functions.
2. The mean square errors of the E-Bayesian estimations of parameter λ and R(t) are smaller than those of the Bayesian estimations. Therefore, the efficiency of the E-Bayesian method is higher in the sense of a smaller MSE.
3. The MSEs of estimations under an SE loss function are less than those based on a LINEX loss function. Thus, the SE loss function is more efficient to generate estimates.
4. As (n,(r,k),T) increases, the MSE of the estimate decreases, and the average interval length of CRIs reduces. To conclude, the performance of estimates will improve with the size of the sample increases.

## 6. Illustrative Example

In order to clarify the algorithms and examine the accuracy and robustness of the theoretical results discussed above, we analyze a real data set of the number of cycles to failure from the electrical devices given by [22]. The authors in [4] divided each data point by 1000 in order to compute effectively, and they tested and concluded that the hazard rate of this data set was bathtub-shaped. Table 10 illustrates the electrical lifetime data in detail in which the unit of the data is the number of cycles.

When analyzing this data set under Type-I GHCS, we assume that (n,(r,k),T)=(60,(18,15),2) and evaluate estimates against SE and LINEX loss functions.

Under this assumption, $X_{k:n}=X_{15:60}=0.917<T$.

Thus, the terminated time will be $T_{*}=\min\left(X_{r:n}=1.064,T=2\right)=1.064$, and the number of failures is 18. According to (8), the MLE of parameter λ is obtained as: $\hat{\lambda }=\frac{n}{T}=\frac{n}{-\sum_{i=1}^{r}W_{i}}=0.3870718$.

According to Table 11 and Table 12, the good performance of Bayesian and E-Bayesian estimations against different loss functions can be certified. The estimates are consistent with the real data sets. Similarly, we can determine that the MSEs of parameter λ and R(t) based on the E-Bayesian approach are smaller than those under the Bayesian approach. It is also more efficient to evaluate estimates against the SE loss function. These are consistent with the statistical inference and numerical simulation results. Thus, it is reasonable to conclude that the theoretical results discussed above are accurate and robust.

**Figure 6:**
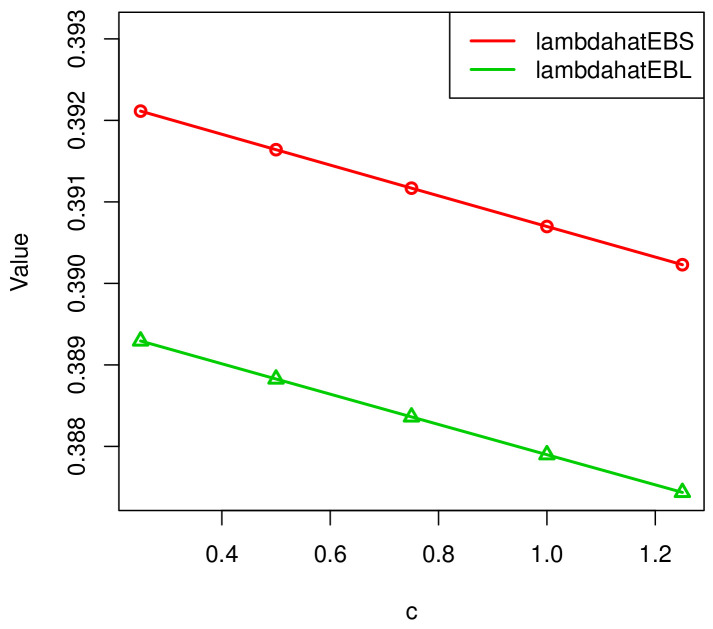
The relationship between *c* and ${\hat{\lambda }}_{EBS}$, ${\hat{\lambda }}_{EBL}$.

**Figure 7:**
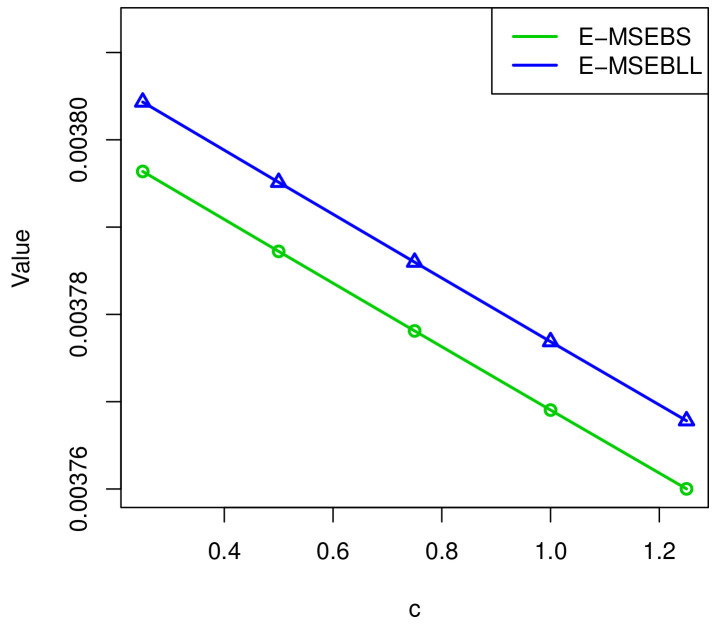
The relationship between *c* and $E-MSE({\hat{\lambda }}_{EBS})$, $E-MSE({\hat{\lambda }}_{EBL})$.

Figure 6 and Figure 7 show the relationship between *c*, $\hat{\lambda }$, and $MSE(\hat{\lambda })$. As *c* increases, the value of ${\hat{\lambda }}_{EB}$ decreases and the value of $E-MSE(\hat{\lambda })$ reduces.

## 7. Conclusions

The bathtub-shaped distribution is crucial in mechanical and electronic research. In addition, it is more efficient to estimate parameters under Type-I GHCS for product testing situations in practice, as this could save the time of testing and the cost resulting from failures of units.

In this article, in order to make estimations under a bathtub-shaped distribution, the Bayesian and E-Bayesian methods were introduced. According to Bayesian theory, we could generate statistical inference from the prior information. With the assumption that the prior distribution follows a gamma distribution, we could derive the estimates of parameter and reliability functions under different loss functions.

We presented the MCMC method in simulation and with a real lifetime example of electronic data to illustrate the statistical inferences discussed above. Under different sample sizes, parameter values and loss functions, we observed that the E-Bayesian method and SE loss function were more efficient in terms of the mean square error. Our study is useful and efficient for experimenters to examine the quality of industrial products. Additionally, this research can be further developed to address practical problems based on multiple censoring schemes.  

## Figures and Tables

**Table 1:** Estimations of λ=4.37 under an SE loss function when β=1,h=1.5,c=1,a=0.6119, and b=0.1523.

*n*	50		80		120	
(r,k)	(30,40)		(60,40)		(90,60)	
*T*	0.2	0.4	0.2	0.4	0.2	0.4
${\hat{\lambda }}_{BS}$	4.4335	4.4768	4.3911	4.4303	4.3831	4.4187
$MSE({\hat{\lambda }}_{BS})$	0.6119	0.5065	0.3862	0.3293	0.2570	0.2179
min	1.7563	2.5385	2.9202	2.9999	3.4522	3.5749
max	7.1331	6.2912	5.9283	5.7578	5.3641	5.2460
length	5.3767	3.7527	3.0081	2.7579	1.9120	1.6711
*n*	50		80		120	
(r,k)	(30,40)		(60,40)		(90,60)	
*T*	0.2	0.4	0.2	0.4	0.2	0.4
${\hat{\lambda }}_{EBS}$	4.3613	4.4168	4.3447	4.3908	4.3519	4.3923
$E-MSE({\hat{\lambda }}_{EBS})$	0.5942	0.4943	0.3790	0.3240	0.2538	0.2155
min	2.1555	2.5336	2.9889	3.1245	3.3145	3.5509
max	6.3399	5.8610	5.5518	5.4765	5.3042	5.0982
length	4.1844	3.3274	2.5628	2.3520	1.9897	1.5473

**Table 2:** Estimations of R(t) under an SE loss function when β=1,h=1.5,c=1,a=0.6119,b=0.1523, and t=0.07.

*n*	50		80		120	
(r,k)	(30,40)		(60,40)		(90,60)	
*T*	0.2	0.4	0.2	0.4	0.2	0.4
$\hat{R}{\left(t\right)}_{BS}$	0.73977	0.73287	0.74154	0.74467	0.74416	0.74291
$MSE(\hat{R}{\left(t\right)}_{BS})$	0.00109	0.00088	0.00057	0.00038	0.00019	0.00012
min	0.61717	0.65334	0.66958	0.67735	0.69564	0.70122
max	0.88796	0.84219	0.82072	0.81631	0.7917	0.78515
length	0.27079	0.18885	0.15113	0.13895	0.09607	0.08394
*n*	50		80		120	
(r,k)	(30,40)		(60,40)		(90,60)	
*T*	0.2	0.4	0.2	0.4	0.2	0.4
$\hat{R}{\left(t\right)}_{EBS}$	0.74913	0.74328	0.74888	0.74962	0.74867	0.74657
$E-MSE(\hat{R}{\left(t\right)}_{EBS})$	0.00096	0.00077	0.00047	0.00033	0.00017	0.00011
min	0.65119	0.67264	0.68686	0.69037	0.69846	0.70826
max	0.864300	0.84247	0.81691	0.80945	0.79911	0.78643
length	0.213100	0.16983	0.13005	0.11908	0.10065	0.07817

**Table 3:** Estimations of λ=4.37 under a LINEX loss function when β=1,h=1.5,c=1,a=0.6119, and b=0.1523.

*n*	50		80		120	
(r,k)	(30,40)		(60,40)		(90,60)	
*T*	0.2	0.4	0.2	0.4	0.2	0.4
${\hat{\lambda }}_{BL}$	4.0436	4.1358	4.1247	4.2005	4.2009	4.2630
$MSE({\hat{\lambda }}_{BL})$	0.7860	0.6341	0.4601	0.3856	0.2911	0.2432
min	1.2176	1.2062	2.2857	2.5972	2.9827	3.2330
max	7.2472	7.137	5.8513	5.6587	5.2302	5.0823
length	6.0296	5.9308	3.5656	3.0615	2.2475	1.8493
*n*	50		80		120	
(r,k)	(30,40)		(60,40)		(90,60)	
*T*	0.2	0.4	0.2	0.4	0.2	0.4
${\hat{\lambda }}_{EBL}$	3.9688	4.0837	4.0831	4.1645	4.1720	4.2382
$E-MSE({\hat{\lambda }}_{EBL})$	0.7590	0.6160	0.4502	0.3785	0.2870	0.2403
min	0.8753	1.5263	2.3519	2.5144	2.9413	3.3233
max	6.3150	5.9493	5.5490	5.6569	5.2711	5.0406
length	5.4397	4.4230	3.1970	3.1425	2.3298	1.7173

**Table 4:** Estimations of R(t) under a LINEX loss function when β=1,h=1.5,c=1,a=0.6119,b=0.1523, and t=0.07.

*n*	50		80		120	
(r,k)	(30,40)		(60,40)		(90,60)	
*T*	0.2	0.4	0.2	0.4	0.2	0.4
$\hat{R}{\left(t\right)}_{BL}$	0.76178	0.75368	0.75179	0.75539	0.75372	0.75066
$MSE(\hat{R}{\left(t\right)}_{BL})$	0.00173	0.00128	0.00073	0.00055	0.00028	0.00016
min	0.61242	0.61700	0.67308	0.68191	0.70197	0.70903
max	0.92092	0.92163	0.85672	0.83885	0.81725	0.80353
length	0.30850	0.30463	0.18364	0.15694	0.11529	0.09450
*n*	50		80		120	
(r,k)	(30,40)		(60,40)		(90,60)	
*T*	0.2	0.4	0.2	0.4	0.2	0.4
$\hat{R}{\left(t\right)}_{EBL}$	0.77050	0.76209	0.75857	0.76036	0.75788	0.75433
$E-MSE(\hat{R}{\left(t\right)}_{EBL})$	0.00141	0.00107	0.00064	0.00047	0.00026	0.00015
min	0.65229	0.66863	0.68699	0.68199	0.70003	0.71103
max	0.94250	0.90189	0.85289	0.84356	0.81954	0.79863
length	0.29021	0.23325	0.16590	0.16157	0.11952	0.08761

**Table 5:** Estimates of parameter λ=4.37 for different loss functions when β=2,h=1.5,a=0.6119, and b=0.1523.

*n*	(r,k)	*T*	*c*	${\hat{\lambda }}_{\mathrm{BS}}$	$\mathrm{MSE}({\hat{\lambda }}_{\mathrm{BS}})$	${\hat{\lambda }}_{\mathrm{EBS}}$	$E-\mathrm{MSE}({\hat{\lambda }}_{\mathrm{EBS}})$	${\hat{\lambda }}_{\mathrm{BL}}$	$\mathrm{MSE}({\hat{\lambda }}_{\mathrm{BL}})$	${\hat{\lambda }}_{\mathrm{EBL}}$	$E-\mathrm{MSE}({\hat{\lambda }}_{\mathrm{EBL}})$
50	(40,30)	0.2	0.5	4.5025	0.6847	4.4226	0.6627	4.0575	0.9078	3.9910	0.8723
		0.4		4.4863	0.6670	4.4081	0.6462	4.0509	0.8746	3.9856	0.8418
80	(60,40)	0.2		4.4771	0.5060	4.4172	0.4937	4.1365	0.6330	4.0844	0.6150
		0.4		4.4097	0.4456	4.3564	0.4360	4.1060	0.5420	4.0590	0.5286
120	(90,60)	0.2		4.4369	0.3304	4.3972	0.3250	4.2064	0.3870	4.1703	0.3799
		0.4		4.3957	0.2976	4.3598	0.2933	4.1866	0.3427	4.1536	0.3371
50	(40,30)	0.2	1	4.5057	0.6857	4.4257	0.6637	4.0601	0.9093	3.9935	0.8738
		0.4		4.4867	0.6669	4.4085	0.6462	4.0513	0.8748	3.9860	0.8419
80	(60,40)	0.2		4.4645	0.5037	4.4049	0.4916	4.1254	0.6303	4.0735	0.6123
		0.4		4.4041	0.4448	4.3509	0.4353	4.1009	0.5409	4.0540	0.5275
120	(90,60)	0.2		4.4302	0.3291	4.3907	0.3238	4.2005	0.3852	4.1646	0.3781
		0.4		4.3878	0.2969	4.3519	0.2926	4.1791	0.3417	4.1462	0.3362
50	(40,30)	0.2	1.5	4.4795	0.6779	4.4003	0.6563	4.0386	0.8973	3.9726	0.8624
		0.4		4.4829	0.6657	4.4049	0.6450	4.0484	0.8729	3.9831	0.8401
80	(60,40)	0.2		4.4681	0.5038	4.4085	0.4917	4.1289	0.6298	4.0770	0.6119
		0.4		4.4199	0.4470	4.3665	0.4374	4.1152	0.5440	4.0681	0.5305
120	(90,60)	0.2		4.4341	0.3298	4.3945	0.3245	4.2040	0.3861	4.1680	0.3791
		0.4		4.4051	0.2986	4.3691	0.2943	4.1953	0.3440	4.1622	0.3384

**Table 6:** Estimates of parameter λ=4.37 for different loss functions when β=3,h=1.5,a=0.6119, and b=0.1523.

*n*	(r,k)	*T*	*c*	${\hat{\lambda }}_{\mathrm{BS}}$	$\mathrm{MSE}({\hat{\lambda }}_{\mathrm{BS}})$	${\hat{\lambda }}_{\mathrm{EBS}}$	$E-\mathrm{MSE}({\hat{\lambda }}_{\mathrm{EBS}})$	${\hat{\lambda }}_{\mathrm{BL}}$	$\mathrm{MSE}({\hat{\lambda }}_{\mathrm{BL}})$	${\hat{\lambda }}_{\mathrm{EBL}}$	$E-\mathrm{MSE}({\hat{\lambda }}_{\mathrm{EBL}})$
50	(40,30)	0.2	0.5	4.5097	0.6869	4.4295	0.6648	4.0633	0.9114	3.9966	0.8757
		0.4		4.4996	0.6837	4.4198	0.6618	4.0552	0.9063	3.9887	0.8709
80	(60,40)	0.2		4.4523	0.5004	4.3930	0.4884	4.1152	0.6249	4.0637	0.6071
		0.4		4.4692	0.5041	4.4095	0.4920	4.1298	0.6303	4.0779	0.6123
120	(90,60)	0.2		4.4306	0.3292	4.3911	0.3239	4.2009	0.3854	4.1649	0.3783
		0.4		4.4220	0.3280	4.3826	0.3227	4.1931	0.3837	4.1573	0.3767
50	(40,30)	0.2	1	4.5168	0.6895	4.4363	0.6673	4.0688	0.9161	4.0020	0.8802
		0.4		4.4953	0.6826	4.4156	0.6607	4.0515	0.9044	3.9852	0.8691
80	(60,40)	0.2		4.4642	0.5032	4.4046	0.4910	4.1254	0.6290	4.0736	0.6111
		0.4		4.4663	0.5038	4.4066	0.4916	4.1271	0.6301	4.0752	0.6121
120	(90,60)	0.2		4.4228	0.3282	4.3834	0.3229	4.1938	0.3840	4.1580	0.3770
		0.4		4.4383	0.3306	4.3986	0.3252	4.2077	0.3873	4.1716	0.3802
50	(40,30)	0.2	1.5	4.5030	0.6847	4.4231	0.6628	4.0579	0.9078	3.9914	0.8723
		0.4		4.4950	0.6821	4.4154	0.6603	4.0515	0.9033	3.9852	0.8681
80	(60,40)	0.2		4.4738	0.5053	4.4140	0.4931	4.1336	0.6321	4.0816	0.6140
		0.4		4.4621	0.5029	4.4025	0.4908	4.1234	0.6289	4.0717	0.6110
120	(90,60)	0.2		4.4304	0.3292	4.3908	0.3239	4.2007	0.3853	4.1647	0.3782
		0.4		4.4410	0.3310	4.4013	0.3257	4.2101	0.3879	4.1740	0.3808

**Table 7:** Estimates of parameter λ=2.5 for different loss functions when β=1,h=1.5,a=0.6119, and b=0.1523.

*n*	(r,k)	*T*	*c*	${\hat{\lambda }}_{\mathrm{BS}}$	$\mathrm{MSE}({\hat{\lambda }}_{\mathrm{BS}})$	${\hat{\lambda }}_{\mathrm{EBS}}$	$E-\mathrm{MSE}({\hat{\lambda }}_{\mathrm{EBS}})$	${\hat{\lambda }}_{\mathrm{BL}}$	$\mathrm{MSE}({\hat{\lambda }}_{\mathrm{BL}})$	${\hat{\lambda }}_{\mathrm{EBL}}$	$E-\mathrm{MSE}({\hat{\lambda }}_{\mathrm{EBL}})$
50	(40,30)	0.2	0.5	2.6002	0.2284	2.5691	0.2237	2.443	0.2564	2.415	0.2505
		0.4		2.5623	0.1843	2.5368	0.1812	2.4334	0.2021	2.41	0.1984
80	(60,40)	0.2		2.5758	0.166	2.5529	0.1635	2.459	0.1808	2.4378	0.1778
		0.4		2.538	0.1144	2.522	0.1131	2.4559	0.1214	2.4408	0.1201
120	(90,60)	0.2		2.549	0.1087	2.5339	0.1076	2.4709	0.1151	2.4565	0.1139
		0.4		2.5278	0.0753	2.5172	0.0748	2.473	0.0784	2.4627	0.0779
50	(40,30)	0.2	1	2.5973	0.2279	2.5663	0.2232	2.4405	0.2558	2.4125	0.25
		0.4		2.5545	0.1831	2.5291	0.1801	2.4263	0.2007	2.4031	0.1971
80	(60,40)	0.2		2.5708	0.1654	2.5479	0.1629	2.4543	0.1801	2.4332	0.1772
		0.4		2.5338	0.114	2.5178	0.1128	2.452	0.121	2.4369	0.1196
120	(90,60)	0.2		2.5494	0.1087	2.5342	0.1076	2.4712	0.1151	2.4569	0.1139
		0.4		2.529	0.0754	2.5184	0.0749	2.4741	0.0785	2.4639	0.0779
50	(40,30)	0.2	1.5	2.6051	0.2291	2.5739	0.2244	2.4474	0.2573	2.4194	0.2514
		0.4		2.5485	0.1826	2.5232	0.1796	2.4207	0.2001	2.3975	0.1965
80	(60,40)	0.2		2.5743	0.1658	2.5514	0.1633	2.4575	0.1806	2.4364	0.1777
		0.4		2.5402	0.1144	2.5243	0.1132	2.4581	0.1215	2.443	0.1202
120	(90,60)	0.2		2.5516	0.1088	2.5364	0.1077	2.4733	0.1153	2.4589	0.1141
		0.4		2.5217	0.075	2.5111	0.0745	2.467	0.0781	2.4569	0.0776

**Table 8:** Estimates of parameter λ=2.5 for different loss functions when β=2,h=1.5,a=0.6119, and b=0.1523.

*n*	(r,k)	*T*	*c*	${\hat{\lambda }}_{\mathrm{BS}}$	$\mathrm{MSE}({\hat{\lambda }}_{\mathrm{BS}})$	${\hat{\lambda }}_{\mathrm{EBS}}$	$E-\mathrm{MSE}({\hat{\lambda }}_{\mathrm{EBS}})$	${\hat{\lambda }}_{\mathrm{BL}}$	$\mathrm{MSE}({\hat{\lambda }}_{\mathrm{BL}})$	${\hat{\lambda }}_{\mathrm{EBL}}$	$E-\mathrm{MSE}({\hat{\lambda }}_{\mathrm{EBL}})$
50	(40,30)	0.2	0.5	2.6032	0.2288	2.5720	0.2240	2.4457	0.2568	2.4177	0.2510
		0.4		2.6071	0.2298	2.5758	0.2250	2.4490	0.2583	2.4209	0.2524
80	(60,40)	0.2		2.5813	0.1683	2.5581	0.1657	2.4629	0.1838	2.4415	0.1807
		0.4		2.5817	0.1682	2.5586	0.1656	2.4634	0.1835	2.4420	0.1805
120	(90,60)	0.2		2.5466	0.1088	2.5315	0.1077	2.4684	0.1154	2.4540	0.1141
		0.4		2.5556	0.1096	2.5403	0.1085	2.4768	0.1163	2.4623	0.1150
50	(40,30)	0.2	1	2.5997	0.2283	2.5686	0.2236	2.4426	0.2562	2.4146	0.2504
		0.4		2.6085	0.2299	2.5773	0.2252	2.4503	0.2583	2.4222	0.2524
80	(60,40)	0.2		2.5714	0.1670	2.5484	0.1645	2.4539	0.1823	2.4326	0.1792
		0.4		2.5718	0.1670	2.5487	0.1644	2.4543	0.1821	2.4330	0.1791
120	(90,60)	0.2		2.5544	0.1095	2.5391	0.1084	2.4756	0.1162	2.4612	0.1149
		0.4		2.5541	0.1094	2.5389	0.1083	2.4754	0.1160	2.4610	0.1148
50	(40,30)	0.2	1.5	2.5966	0.2279	2.5655	0.2232	2.4397	0.2560	2.4118	0.2501
		0.4		2.6042	0.2294	2.5730	0.2246	2.4464	0.2578	2.4183	0.2519
80	(60,40)	0.2		2.5766	0.1678	2.5535	0.1652	2.4586	0.1831	2.4373	0.1801
		0.4		2.5842	0.1687	2.5610	0.1661	2.4655	0.1843	2.4441	0.1812
120	(90,60)	0.2		2.5479	0.1089	2.5327	0.1078	2.4696	0.1155	2.4552	0.1142
		0.4		2.5490	0.1090	2.5339	0.1079	2.4707	0.1156	2.4563	0.1143

**Table 9:** Estimates of parameter λ=2.5 for different loss functions when β=3,h=1.5,a=0.6119, and b=0.1523.

*n*	(r,k)	*T*	*c*	${\hat{\lambda }}_{\mathrm{BS}}$	$\mathrm{MSE}({\hat{\lambda }}_{\mathrm{BS}})$	${\hat{\lambda }}_{\mathrm{EBS}}$	$E-\mathrm{MSE}({\hat{\lambda }}_{\mathrm{EBS}})$	${\hat{\lambda }}_{\mathrm{BL}}$	$\mathrm{MSE}({\hat{\lambda }}_{\mathrm{BL}})$	${\hat{\lambda }}_{\mathrm{EBL}}$	$E-\mathrm{MSE}({\hat{\lambda }}_{\mathrm{EBL}})$
50	(40,30)	0.2	0.5	2.6063	0.2295	2.5750	0.2247	2.4484	0.2578	2.4203	0.2519
		0.4		2.6006	0.2286	2.5695	0.2239	2.4433	0.2568	2.4153	0.2509
80	(60,40)	0.2		2.5750	0.1675	2.5519	0.1649	2.4572	0.1827	2.4359	0.1797
		0.4		2.5793	0.1681	2.5562	0.1655	2.4611	0.1835	2.4398	0.1805
120	(90,60)	0.2		2.5610	0.1101	2.5456	0.1090	2.4818	0.1168	2.4673	0.1155
		0.4		2.5565	0.1097	2.5412	0.1086	2.4776	0.1163	2.4632	0.1151
50	(40,30)	0.2	1	2.6065	0.2296	2.5753	0.2249	2.4485	0.2581	2.4204	0.2522
		0.4		2.6147	0.2311	2.5833	0.2263	2.4558	0.2598	2.4275	0.2538
80	(60,40)	0.2		2.5758	0.1675	2.5527	0.1650	2.4579	0.1828	2.4366	0.1798
		0.4		2.5810	0.1682	2.5579	0.1656	2.4627	0.1836	2.4413	0.1806
120	(90,60)	0.2		2.5532	0.1094	2.5379	0.1083	2.4746	0.1160	2.4601	0.1147
		0.4		2.5505	0.1091	2.5353	0.1080	2.4721	0.1157	2.4577	0.1145
50	(40,30)	0.2	1.5	2.6066	0.2296	2.5753	0.2249	2.4485	0.2581	2.4204	0.2522
		0.4		2.6105	0.2306	2.5792	0.2258	2.4519	0.2593	2.4237	0.2534
80	(60,40)	0.2		2.5752	0.1673	2.5522	0.1648	2.4575	0.1826	2.4362	0.1795
		0.4		2.5788	0.1680	2.5557	0.1654	2.4607	0.1833	2.4393	0.1803
120	(90,60)	0.2		2.5443	0.1086	2.5291	0.1075	2.4662	0.1151	2.4518	0.1139
		0.4		2.5531	0.1094	2.5378	0.1083	2.4745	0.1160	2.4600	0.1147

**Table 10:** Real data set of 60 observations of electrical appliances.

0.014	0.034	0.059	0.061	0.069	0.080	0.123	0.142	0.165	0.210
0.381	0.464	0.479	0.556	0.574	0.839	0.917	0.969	0.991	1.064
1.088	1.091	1.174	1.270	1.275	1.355	1.397	1.477	1.578	1.649
1.702	1.893	1.932	2.001	2.161	2.292	2.326	2.337	2.628	2.785
2.811	2.886	2.993	3.122	3.248	3.715	3.790	3.857	3.912	4.100
4.106	4.116	4.315	4.510	4.580	5.267	5.299	5.583	6.065	9.701

**Table 11:** The results of estimates of λ for the real data set.

*c*	${\hat{\lambda }}_{\mathrm{BS}}$	$\mathrm{MSE}({\hat{\lambda }}_{\mathrm{BS}})$	${\hat{\lambda }}_{\mathrm{EBS}}$	$E-\mathrm{MSE}({\hat{\lambda }}_{\mathrm{EBS}})$	${\hat{\lambda }}_{\mathrm{BL}}$	$\mathrm{MSE}({\hat{\lambda }}_{\mathrm{BL}})$	${\hat{\lambda }}_{\mathrm{EBL}}$	$E-\mathrm{MSE}({\hat{\lambda }}_{\mathrm{EBL}})$
0.25	0.3931	0.003801	0.3921	0.003796	0.3903	0.003812	0.3893	0.003804
0.50	0.3931	0.003801	0.3916	0.003787	0.3903	0.003812	0.3888	0.003795
1.00	0.3931	0.003801	0.3912	0.003778	0.3903	0.003812	0.3884	0.003785
1.25	0.3931	0.003801	0.3907	0.003769	0.3903	0.003812	0.3879	0.003776
1.50	0.3931	0.003801	0.3902	0.003759	0.3903	0.003812	0.3874	0.003771

**Table 12:** The results of estimates of R(t) for the real data set.

c	${R(t)}_{\mathrm{BS}}$	${R(t)}_{\mathrm{BL}}$	${R(t)}_{\mathrm{EBS}}$	${R(t)}_{\mathrm{EBL}}$
0.25	0.730308	0.731945	0.730892	0.732531
0.50	0.730308	0.731945	0.731185	0.732823
1.00	0.730308	0.731945	0.731419	0.733058
1.25	0.730308	0.731945	0.731711	0.733351
1.50	0.730308	0.731945	0.732004	0.733644

**Figure A1:**
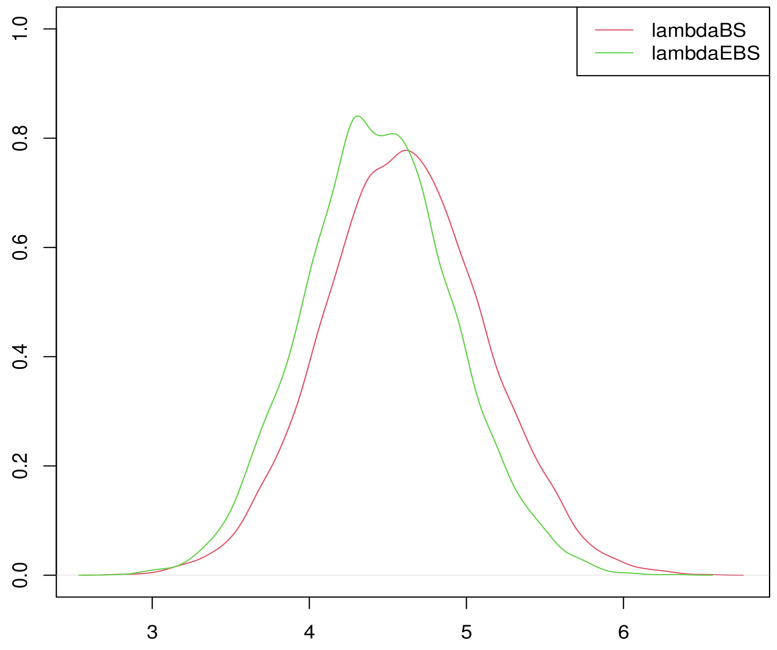
The sample density of λ when N=10,000.

**Figure A2:**
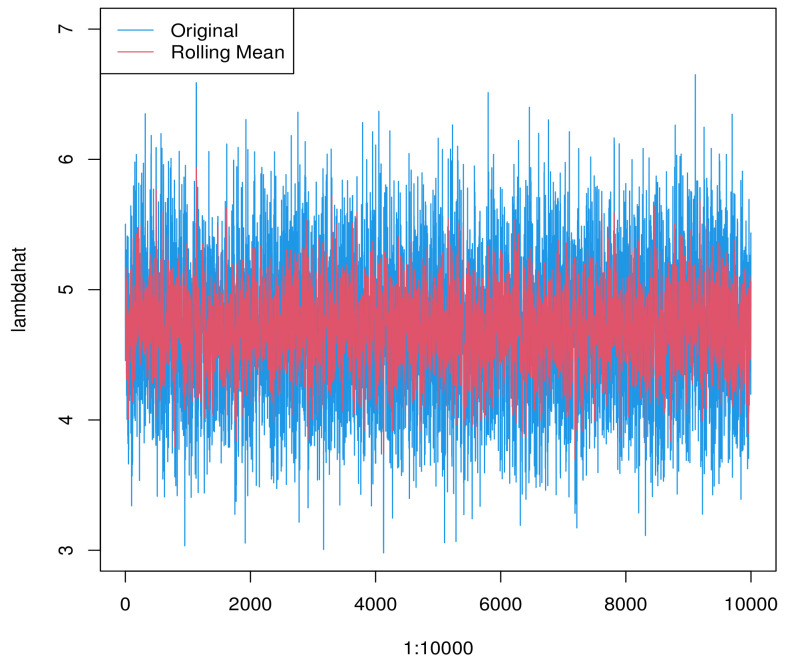
The sequence of white noise in the draws.

**Figure A3:**
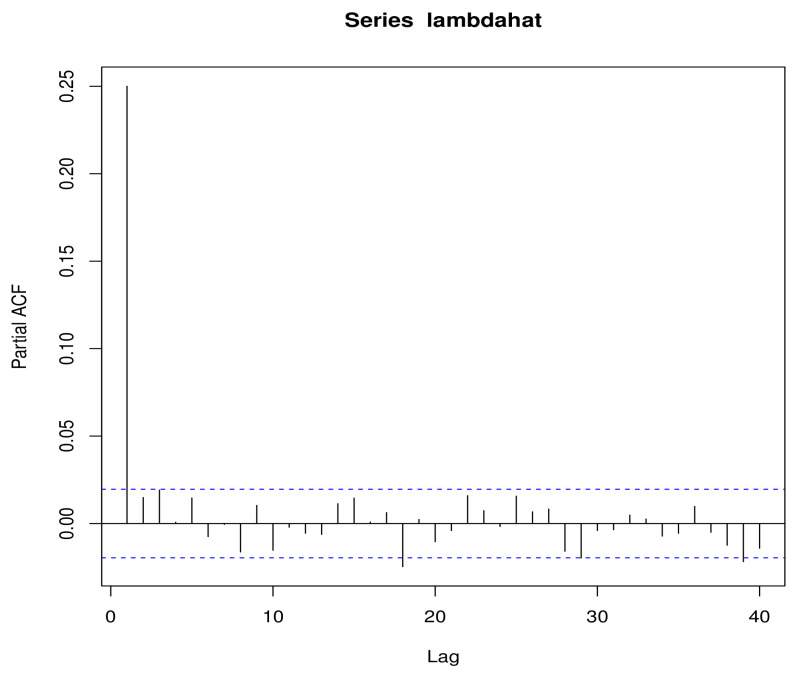
The autocorrelation coefficient of λ in the draws.

**Figure A4:**
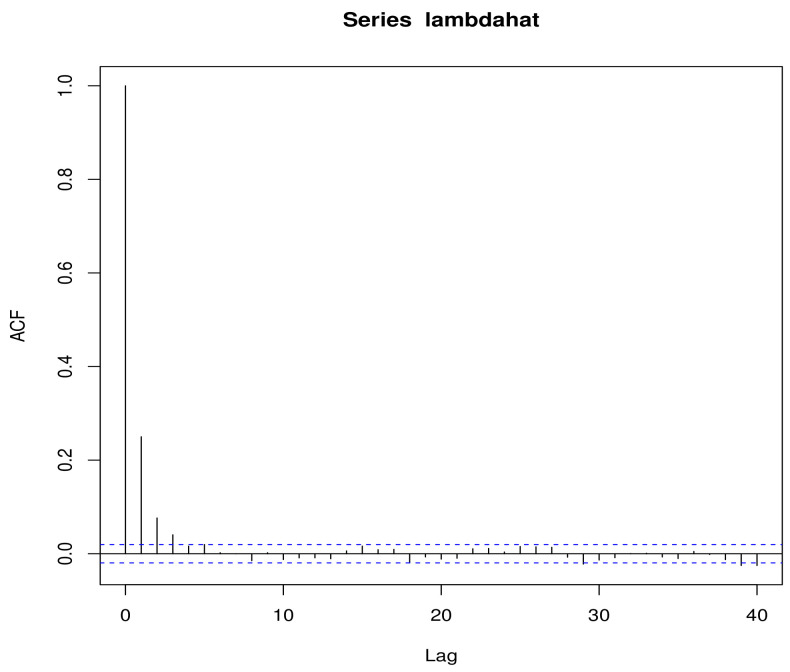
The partial autocorrelation coefficient of λ in the draws.

## Data Availability

The data presented in this study are openly available in [22].

## Appendix A. MCMC Outputs

According to Figure A1, we can determine than the sample density of λ approximately obeys the law of normal distribution. Figure A2, Figure A3 and Figure A4 show the stationarity of the Markov Chain.

## Appendix B. The Robustness of the Simulation with Different h

Table A1 and Table A2 show that the difference of estimations between different *h* values is relatively small, and the results still follow the statistical inference discussed above. Therefore, the robustness of the simulations with different *h* values is verified.

**Table A1:** Estimates of parameter λ=2.5 for different loss functions when β=1,h=3,a=0.6119, and b=0.1523.

*n*	(r,k)	*T*	*c*	${\hat{\lambda }}_{\mathrm{BS}}$	$\mathrm{MSE}({\hat{\lambda }}_{\mathrm{BS}})$	${\hat{\lambda }}_{\mathrm{EBS}}$	$E-\mathrm{MSE}({\hat{\lambda }}_{\mathrm{EBS}})$	${\hat{\lambda }}_{\mathrm{BL}}$	$\mathrm{MSE}({\hat{\lambda }}_{\mathrm{BL}})$	${\hat{\lambda }}_{\mathrm{EBL}}$	$E-\mathrm{MSE}({\hat{\lambda }}_{\mathrm{EBL}})$
50	(40,30)	0.2	0.5	2.6054	0.2292	2.5742	0.2245	2.3134	0.3251	2.2878	0.3166
		0.4		2.5541	0.1832	2.5287	0.1801	2.3135	0.2450	2.2920	0.2399
80	(60,40)	0.2		2.5796	0.1665	2.5567	0.1640	2.3585	0.2193	2.3387	0.2152
		0.4		2.5377	0.1142	2.5217	0.1130	2.3804	0.1402	2.3661	0.1384
120	(90,60)	0.2		2.5481	0.1085	2.5329	0.1074	2.3981	0.1324	2.3844	0.1308
		0.4		2.5221	0.0751	2.5115	0.0745	2.4158	0.0867	2.4060	0.0860
50	(40,30)	0.2	1	2.6092	0.2299	2.5779	0.2251	2.3164	0.3262	2.2908	0.3177
		0.4		2.5552	0.1833	2.5298	0.1802	2.3145	0.2451	2.2930	0.2401
80	(60,40)	0.2		2.5645	0.1646	2.5418	0.1621	2.3457	0.2163	2.3261	0.2123
		0.4		2.5385	0.1143	2.5226	0.1131	2.3812	0.1403	2.3668	0.1385
120	(90,60)	0.2		2.5523	0.1089	2.5371	0.1078	2.4018	0.1329	2.3881	0.1313
		0.4		2.5243	0.0752	2.5137	0.0747	2.4179	0.0869	2.4080	0.0862
50	(40,30)	0.2	1.5	2.6017	0.2285	2.5706	0.2238	2.3105	0.3237	2.2850	0.3153
		0.4		2.5570	0.1835	2.5316	0.1805	2.3161	0.2456	2.2945	0.2406
80	(60,40)	0.2		2.5778	0.1663	2.5548	0.1638	2.3569	0.2190	2.3372	0.2150
		0.4		2.5392	0.1144	2.5233	0.1132	2.3818	0.1404	2.3675	0.1386
120	(90,60)	0.2		2.5416	0.1080	2.5266	0.1069	2.3924	0.1316	2.3788	0.1300
		0.4		2.5258	0.0752	2.5153	0.0747	2.4193	0.0869	2.4095	0.0863

**Table A2:** Estimates of parameter λ=2.5 for different loss functions when β=1,h=0.5,a=0.6119, and b=0.1523.

*n*	(r,k)	*T*	*c*	${\hat{\lambda }}_{\mathrm{BS}}$	$\mathrm{MSE}({\hat{\lambda }}_{\mathrm{BS}})$	${\hat{\lambda }}_{\mathrm{EBS}}$	$E-\mathrm{MSE}({\hat{\lambda }}_{\mathrm{EBS}})$	${\hat{\lambda }}_{\mathrm{BL}}$	$\mathrm{MSE}({\hat{\lambda }}_{\mathrm{BL}})$	${\hat{\lambda }}_{\mathrm{EBL}}$	$E-\mathrm{MSE}({\hat{\lambda }}_{\mathrm{EBL}})$
50	(40,30)	0.2	0.5	2.6014	0.2285	2.5703	0.2238	2.5460	0.2320	2.5160	0.2272
		0.4		2.5572	0.1835	2.5318	0.1804	2.5124	0.1857	2.4877	0.1825
80	(60,40)	0.2		2.5721	0.1656	2.5493	0.1631	2.5316	0.1674	2.5094	0.1648
		0.4		2.5373	0.1142	2.5213	0.1130	2.5092	0.1150	2.4935	0.1138
120	(90,60)	0.2		2.5559	0.1092	2.5407	0.1081	2.5290	0.1100	2.5140	0.1088
		0.4		2.5281	0.0754	2.5175	0.0748	2.5095	0.0757	2.4990	0.0752
50	(40,30)	0.2	1	2.6168	0.2312	2.5854	0.2265	2.5607	0.2348	2.5304	0.2299
		0.4		2.5595	0.1837	2.5341	0.1806	2.5146	0.1859	2.4900	0.1827
80	(60,40)	0.2		2.5747	0.1659	2.5519	0.1634	2.5342	0.1677	2.5119	0.1651
		0.4		2.5361	0.1141	2.5201	0.1129	2.5079	0.1150	2.4923	0.1137
120	(90,60)	0.2		2.5527	0.1089	2.5376	0.1078	2.5259	0.1097	2.5110	0.1086
		0.4		2.5216	0.0750	2.5111	0.0745	2.5030	0.0754	2.4926	0.0748
50	(40,30)	0.2	1.5	2.6069	0.2297	2.5757	0.2249	2.5512	0.2332	2.5211	0.2283
		0.4		2.5543	0.1831	2.5290	0.1801	2.5096	0.1853	2.4850	0.1821
80	(60,40)	0.2		2.5765	0.1661	2.5536	0.1636	2.5359	0.1679	2.5136	0.1653
		0.4		2.5309	0.1138	2.5149	0.1126	2.5028	0.1146	2.4872	0.1134
120	(90,60)	0.2		2.5459	0.1084	2.5308	0.1073	2.5192	0.1091	2.5044	0.1080
		0.4		2.5283	0.0754	2.5177	0.0749	2.5096	0.0757	2.4991	0.0752

## Appendix C. MCMC Method for Two Unknown Parameters

**Algorithm A1** The MCMC algorithm for two unknown parameters.
1: Set the initial value $\lambda^{\left(0\right)}$, $\beta^{\left(0\right)}$ be equal to the MLE $\hat{\lambda }$, MLE $\hat{\beta }$. 2: Set v=1. 3: **repeat** 4: Take $N(\hat{\lambda },Var\left(\hat{\lambda }\right))$ and $N(\hat{\beta },Var\left(\hat{\beta }\right))$ as the proposal distribution and generate $\lambda^{\left(*\right)}$ and $\beta^{\left(*\right)}$ from it at iteration *v*. 5: Obtain the samples under Type-I GHCS from Uniform(0,1) distribution. 6: Compute the acceptance probability: $p_{1}\left(\lambda^{\left(v-1\right)}|\lambda^{\left(*\right)}\right)=\min\left[\frac{\pi^{*}\left(\lambda^{\left(*\right)}|\tilde{x}\right)}{\pi^{*}\left(\lambda^{\left(v-1\right)}|\tilde{x}\right)},1\right]$, $p_{2}\left(\beta^{\left(v-1\right)}|\beta^{\left(*\right)}\right)=\min\left[\frac{\pi^{*}\left(\beta^{\left(*\right)}|\tilde{x}\right)}{\pi^{*}\left(\beta^{\left(v-1\right)}|\tilde{x}\right)},1\right]$. 7: **if**$\;u<p_{1}$ and $u<p_{2}$ **then** 8: $\lambda^{\left(v\right)}=\lambda^{\left(*\right)},\beta^{\left(v\right)}=\beta^{\left(*\right)}$; 9: **else** 10: $\lambda^{\left(v\right)}=\lambda^{\left(v-1\right)},\beta^{\left(v\right)}=\beta^{\left(v-1\right)}$; 11: **end if** 12: Compute the value of $R^{\left(v\right)}\left(t\right)$ as $R^{\left(v\right)}\left(t\right)=\exp\left\{\lambda^{\left(v\right)}\left(1-e^{x^{\beta }}\right)\right\}$. 13: **until** v=N 14: Set $\delta =\frac{N}{10}$ as the burn-in period. 15: Based on an SE loss function, ${\hat{\lambda }}_{BS}=\frac{1}{N-\delta }\sum_{v=\delta +1}^{N}\lambda^{\left(v\right)}$, ${\hat{\beta }}_{BS}=\frac{1}{N-\delta }\sum_{v=\delta +1}^{N}\beta^{\left(v\right)}$, ${\hat{R(t)}}_{BS}=\frac{1}{N-\delta }\sum_{v=\delta +1}^{N}R^{\left(v\right)}\left(t\right)$. 16: Based on a LINEX loss function, ${\hat{\lambda }}_{BL}=-\frac{1}{h}\ln\left[\frac{1}{N-\delta }\sum_{v=\delta +1}^{N}e^{-h\lambda^{\left(v\right)}}\right]$, ${\hat{\beta }}_{BL}=-\frac{1}{h}\ln\left[\frac{1}{N-\delta }\sum_{v=\delta +1}^{N}e^{-h\beta^{\left(v\right)}}\right]$, ${\hat{R(t)}}_{BL}=-\frac{1}{h}\ln\left[\frac{1}{N-\delta }\sum_{v=\delta +1}^{N}e^{-hR^{\left(v\right)}\left(t\right)}\right]$. 17: Obtain $a_{1},a_{2},b_{1},$ and $b_{2}$ from $Beta(u_{1},v_{1})$, $Beta(u_{2},v_{2})$, $U(u_{1},v_{1})$, and $U(u_{2},v_{2})$, respectively. Based on steps 15–16, (42)–(45), we can obtain the E-Bayesian estimations.
